# Loss of Gre factors leads to phenotypic heterogeneity and cheating in *Escherichia coli* populations under nitric oxide stress

**DOI:** 10.1128/mbio.02229-24

**Published:** 2024-09-09

**Authors:** Darshan M. Sivaloganathan, Xuanqing Wan, Gabrielle Leon, Mark P. Brynildsen

**Affiliations:** 1 Program in Quantitative and Computational Biology, Princeton University, Princeton, New Jersey, USA; 2 Department of Chemical and Biological Engineering, Princeton University, Princeton, New Jersey, USA; Racah Institute of Physics and the Harvey, The Hebrew University of Jerusalem, Jerusalem, Israel; Imperial College London, London, United Kingdom

**Keywords:** NO, H_2_O_2_, RNA polymerase, GreA, GreB

## Abstract

Nitric oxide (·NO) is one of the toxic metabolites that bacteria can be exposed to within phagosomes. Gre factors, which are also known as transcript cleavage factors or transcription elongation factors, relieve back-tracked transcription elongation complexes by cleaving nascent RNAs, which allows transcription to resume after stalling. Here we discovered that loss of both Gre factors in *Escherichia coli*, GreA and GreB, significantly compromised ·NO detoxification due to ·NO-induced phenotypic heterogeneity in Δ*greA*Δ*greB* populations, which did not occur in wild-type cultures. Under normal culturing conditions, both wild-type and Δ*greA*Δ*greB* synthesized transcripts uniformly, whereas treatment with ·NO led to bimodal transcript levels in Δ*greA*Δ*greB* that were unimodal in wild-type. Interestingly, exposure to another toxic metabolite of phagosomes, hydrogen peroxide (H_2_O_2_), produced analogous results. Furthermore, we showed that loss of Gre factors led to cheating under ·NO stress where transcriptionally deficient cells benefited from the detoxification activities of the transcriptionally proficient subpopulation. Collectively, these results show that loss of Gre factor activities produces phenotypic heterogeneity under ·NO and H_2_O_2_ stress that can yield cheating between subpopulations.

**IMPORTANCE:**

Toxic metabolite stress occurs in a broad range of contexts that are important to human health, microbial ecology, and biotechnology, whereas Gre factors are highly conserved throughout the bacterial kingdom. Here we discovered that loss of Gre factors in *E. coli* leads to phenotypic heterogeneity under ·NO and H_2_O_2_ stress, which we further show with ·NO results in cheating between subpopulations. Collectively, these data suggest that Gre factors play a role in coping with toxic metabolite stress, and that loss of Gre factors can produce cheating between neighbors.

## INTRODUCTION

Phagocytes are instrumental in controlling and eliminating infections as they represent the first line of defense mounted against invading microorganisms (1–4). Within phagosomes, bacteria are exposed to a wide array of antimicrobials that work in concert to eliminate them (1, 2). One important antimicrobial produced in these environments is nitric oxide (·NO) (2, 5–7). Many microbes have evolved defense systems to combat the cytotoxic effects of ·NO, and the ability to detoxify ·NO has been linked to the virulence of numerous pathogens (8, 9). For example, *S. typhimurium* strains lacking the ·NO dioxygenase, Hmp, displayed reduced survival in human macrophages compared to wild-type (WT) cells, but similar survival in the presence of an iNOS inhibitor (10). Similarly, loss of ·NO reductase in enterohemorrhagic *E. coli*, through mutation of *norV*, led to a dramatic reduction in survival within human macrophages (11). Many other bacteria, including *Pseudomonas aeruginosa*, *Vibrio cholerae*, and *Mycobacterium tuberculosis*, have been shown to rely on ·NO detoxification to impart their virulence (12–14). Sensitizing pathogens to ·NO by interfering with their defense mechanisms has been suggested to be an appealing approach to antimicrobial therapies, which would be orthogonal to current treatments (8, 9, 13). A deeper understanding of the networks that regulate ·NO metabolism will facilitate the discovery of novel, accessible targets that can interfere with the virulence of diverse pathogens (8, 9).

Previous work has revealed the importance of the global transcriptional regulator, DksA, to *Salmonella* survival both in macrophages and mouse models (15–17). Furthermore, DksA has been shown to play an essential role in the ability of *E. coli* to detoxify ·NO with Hmp (18). DksA is known to bind directly to the secondary channel of RNA polymerase (RNAP) to exert its regulatory functions (19, 20). Interestingly, DksA is a member of a larger class of proteins that collectively compete for access to the secondary channel of RNAP to exert their functions (21–25). Additional secondary channel proteins include Gre factors (also known as transcript cleavage factors or transcription elongation factors), the nucleoside diphosphate kinase regulator Rnk, the conjugative F plasmid regulator TraR, and the antimicrobial peptide microcin J25 (26–29). While recent research has solidified a central role for DksA in bacterial ·NO defenses, the impacts of other RNAP secondary channel regulators on bacterial ·NO responses remain ill-defined.

Gre factors are highly conserved proteins across the bacterial kingdom that alleviate RNAP stalling and promote transcript elongation (27, 30, 31). RNAP stalling is thought to occur as a result of numerous events including abortive initiation, misincorporation of nucleotides, and collisions with DNA-bound protein complexes (32). RNAP stalling often leads to reverse translocation, where the transcription elongation complex (TEC) slides backward in the 3′ to 5′ direction along the nascent RNA transcript, exposing one or more base pairs (32, 33). Backtracked TECs cannot proceed with transcript elongation, and when held in a backtracked position for prolonged periods of time, they can eventually lead to an arrested transcriptional state, and ultimately dissociation of RNAP (32, 33). Gre factors counteract RNAP stalling by facilitating the 3′ cleavage of nascent RNA in backtracked TECs, allowing transcription to resume (30, 32). The importance of Gre factors to pathogenicity has been established for a number of pathogens (34–39), where it has been shown to regulate a diversity of virulence factors (35, 36).

Inspired by previous work on DksA and ·NO stress, the involvement of Gre factors in the pathogenicity of various microbes, and the fact that both act through the secondary channel of RNAP, we investigated the importance of GreA and GreB to ·NO detoxification by *E. coli*. We found that ·NO detoxification was robust to individual deletion of GreA or GreB; however, the double mutant, Δ*greA*Δ*greB*, was significantly impaired. Further analyses revealed that loss of both Gre factors produced phenotypic heterogeneity under ·NO stress, where a subpopulation produced Hmp, the main aerobic ·NO detoxification enzyme, similarly to WT (responders) and another subpopulation failed to do so (non-responders). Heterogeneous responses were observed with an orthogonal expression system under ·NO stress, whereas distributions were unimodal under normal growth conditions. Experiments with fluorescently labeled RNA, fluorescent proteins, and an independent T7 RNAP expression system established that heterogeneity in RNA synthesis was a major determinant of the heterogeneous responses observed and that both depended on *E. coli* RNAP. Interestingly, we found that the stress-induced phenotypic heterogeneity of Δ*greA*Δ*greB* populations was not confined to ·NO but was also observed in the presence of hydrogen peroxide (H_2_O_2_), which is another phagosome antimicrobial. We then used three independent types of experiment to assess whether cheating occurs in ·NO-stressed Δ*greA*Δ*greB* cultures, and the resulting data support the conclusion that non-responders cheat off of responders in ·NO-stressed *E. coli* cultures without Gre factors. Collectively, these data demonstrate that loss of Gre factors leads to phenotypic heterogeneity and cheating in *E. coli* populations under ·NO stress, and suggest that the phenomenon may be shared with other toxic metabolites, such as H_2_O_2_. Overall, this study contributes to the growing understanding of how regulators that bind to the secondary channel of RNAP modulate bacterial defense networks.

## RESULTS

### Loss of GreA and GreB impairs ·NO detoxification

**Fig 1:**
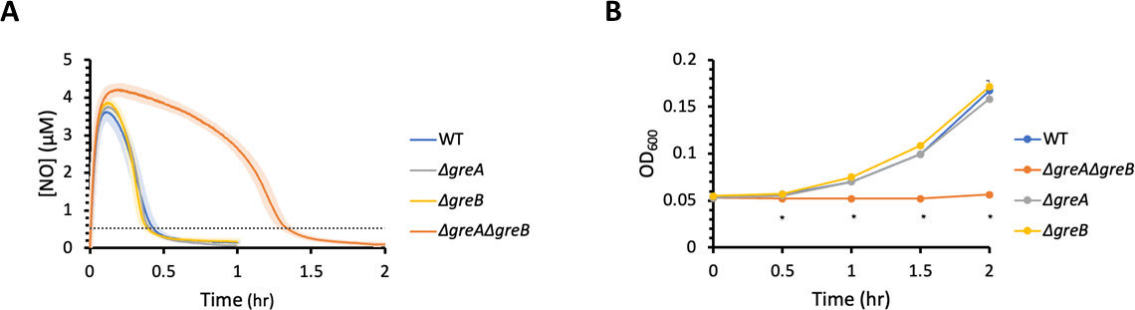
Loss of GreA and GreB significantly impaired ·NO detoxification and growth resumption. Cultures were grown in MOPS (3-(N-Morpholino)propanesulfonic acid) minimal media to mid-exponential phase and inoculated into a bioreactor at an OD_600_ of 0.05. Immediately after, 250 µM of DPTA NONOate was added to the bioreactor. WT (blue), Δ*greA* (gray), Δ*greB* (yellow), Δ*greA*Δ*greB* (orange). (**A**) ·NO concentrations were continuously monitored in the bioreactor. Solid lines represent the means of at least three independent replicates, whereas light shading represents the standard errors of the means. The dashed line indicates a ·NO concentration of 0.5 µM, which was used as the threshold for clearance calculations. (**B**) Samples were removed to measure OD_600_ at indicated time points. Circles represent the means of at least three independent replicates, whereas error bars represent the standard errors of the means. Asterisks indicate statistical significance, at a *P*-value ≤ 0.05, which was assessed by one-way ANOVA and post hoc Tukey HSD tests. Δ*greA*Δ*greB* was significantly different than WT, Δ*greA*, and Δ*greB* at all time points with an asterisk.

To begin to explore the role of Gre factors in ·NO defenses, we constructed single and double deletion mutants of GreA and GreB and measured their abilities to detoxify ·NO compared to WT. Aerobic cultures were grown to mid-exponential phase and treated with 250 µM of the ·NO donor, DPTA NONOate (Fig. 1A). For all cultures, ·NO concentrations rapidly rose and peaked around 4 µM. The clearance times, which we define as the time it takes for [·NO] to fall below 0.5 µM, were approximately 0.4 h for WT, Δ*greA*, and Δ*greB*. However, for Δ*greA*Δ*greB*, the time to eliminate ·NO from cultures was significantly longer at approximately 1.4 h, representing a greater than threefold delay in detoxification. In addition, Δ*greA*Δ*greB* cultures had impaired growth resumption following ·NO stress compared to WT and single deletion mutants (Fig. 1B). Using genetic complementation experiments, we confirmed that the observed phenotype was attributable to loss of both GreA and GreB. Introduction of either *greA* or *greB* when expressed from their native promoters on low-copy plasmids restored ·NO detoxification to WT levels (Fig. S1A). We also observed culturability levels did not vary much from their initial values for both Δ*greA*Δ*greB* and WT under ·NO stress (Fig. S1B), which suggested that cell death was not a contributing factor to the impaired ·NO detoxification observed.

### Δ*greA*Δ*greB* did not display a growth defect in the absence of ·NO stress

**Fig 2:**
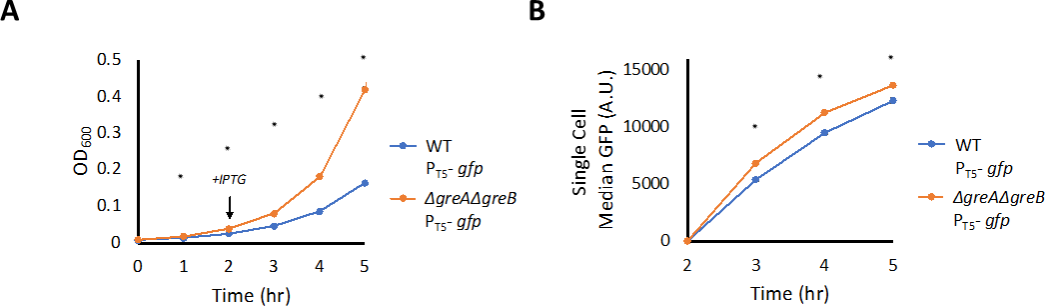
Δ*greA*Δ*greB* displays growth and GFP production advantages in the absence of ·NO stress. WT and Δ*greA*Δ*greB* cultures were inoculated into baffled flasks containing MOPS minimal media at an OD_600_ of 0.01 and incubated at 37°C and 250 RPM. (**A**) Samples were removed to measure OD_600_ at indicated time points. (**B**) After 2 h of incubation, 1 mM IPTG was added to flasks to induce GFP production. Samples were removed and fixed, and median GFP fluorescence was obtained by flow cytometry. WT (blue), Δ*greA*Δ*greB* (orange). Circles represent the means of at least three independent replicates. Error bars represent the standard errors of the means. Asterisks indicate statistical significance, at a *P*-value ≤ 0.05, which was assessed by *t*-tests.

Given the impairment in ·NO detoxification, we considered whether Δ*greA*Δ*greB* in general suffered from growth issues or had difficulty with gene expression since the main determinant of ·NO clearance time in aerobic cultures is *de novo* synthesis of a defense system, Hmp (18, 40, 41). To investigate this, we compared the growth of both WT and Δ*greA*Δ*greB* cultures harboring an isopropyl β-D-1- thiogalactopyranoside (IPTG)-inducible, GFP reporter plasmid. Notably, Δ*greA*Δ*greB* cultures grew significantly faster from lag to mid-exponential phase than WT in the minimal media used here (Fig. 2A), and GFP was produced to significantly higher levels in Δ*greA*Δ*greB* than WT (Fig. 2B; Fig. S1C and D). The growth advantage depicted in Fig. 2 was not dependent on the GFP reporter plasmid (Fig. S1E), and GFP fluorescence was negligible in the absence of the GFP reporter plasmid and IPTG (Fig. S1F). These results demonstrated that during normal, non-stressed growth in minimal media, Δ*greA*Δ*greB* grew faster and expressed protein faster than WT, which suggested that impairments in ·NO detoxification did not originate from general growth or gene expression issues with the mutant.

### Δ*greA*Δ*greB* exposed to ·NO displayed bimodal GFP expression from the *hmp* promoter

In *E. coli*, the enzyme Hmp detoxifies the majority of ·NO under aerobic conditions, and previous work has established that its expression is largely induced *de novo* in response to ·NO (18, 40, 42). To confirm that similar circumstances govern ·NO detoxification under the conditions used here, we deleted *hmp* from WT and Δ*greA*Δ*greB* and measured ·NO consumption. Notably, Δ*hmp* and Δ*greA*Δ*greB*Δ*hmp* cultures both failed to detoxify NO appreciably (Fig. S1G), which suggested that the difference observed between the strains required *hmp*. To assess the importance of *de novo* protein expression under ·NO stress, we treated WT and Δ*greA*Δ*greB* with 100 µg/mL chloramphenicol (CAM) prior to and during ·NO treatment, and observed negligible ·NO detoxification (Fig. S1H). These results established that the difference in ·NO detoxification between WT and Δ*greA*Δ*greB* required Hmp and *de novo* protein expression under ·NO stress.

**Fig 3:**
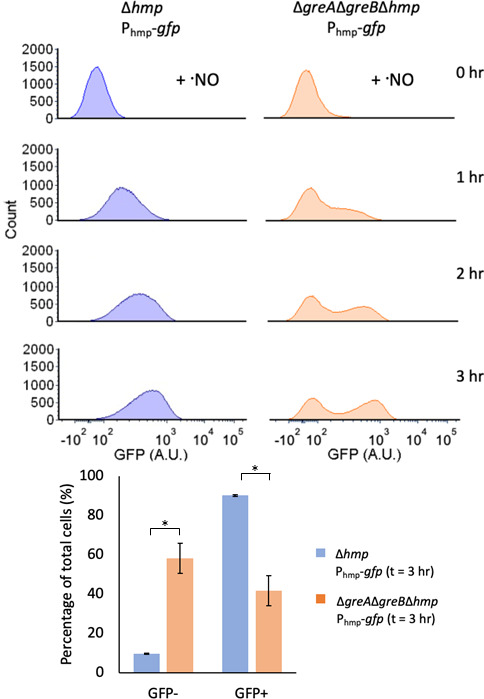
Under ·NO stress, Δ*greA*Δ*greB* populations exhibit bimodal expression from the *hmp* promoter. Cultures were grown in MOPS minimal media to mid-exponential phase and inoculated into a bioreactor at an OD_600_ of 0.05. Immediately after, 250 µM of DPTA NONOate was added. Samples were removed at indicated time points, fixed, and GFP fluorescence was measured by flow cytometry. Histograms represent single-cell GFP fluorescence distributions at indicated time points. The bar chart reflects the percentage of GFP-positive and GFP-negative cells at t = 3 h. Δ*hmp* (blue) and Δ*greA*Δ*greB*Δ*hmp* (orange). Images are representative of at least three biological replicates. Bar charts show the means of at least three independent replicates, whereas error bars represent the standard errors of the means. Asterisks indicate statistical significance, at a *P*-value ≤ 0.05, which was assessed by *t*-tests.

Since Hmp was required to observe differences in ·NO detoxification between WT and Δ*greA*Δ*greB*, we used a transcriptional reporter of *hmp* expression (P_hmp_-*gfp*) in Δ*hmp* and Δ*greA*Δ*greB*Δ*hmp* to monitor GFP production from that promoter during ·NO stress. We opted to use strains devoid of *hmp* here to ensure identical ·NO environments during the assay (Fig. S1G). Interestingly, we observed that Δ*hmp* and Δ*greA*Δ*greB*Δ*hmp* displayed drastically different fluorescence distributions (Fig. 3). Populations of Δ*hmp* expressed GFP in a unimodal fashion that increased in magnitude over time, culminating in over 90% of cells synthesizing GFP over t = 0 levels by 3 h. By contrast, Δ*greA*Δ*greB*Δ*hmp* populations expressed GFP in a bimodal fashion, in which one subpopulation of cells did not increase expression of GFP over time, whereas the other subpopulation expressed GFP at levels similar to Δ*hmp*. After 3 h of ·NO stress, only 42% of Δ*greA*Δ*greB*Δ*hmp* cells produced GFP above t = 0 levels, which was significantly less than that of Δ*hmp*. Complementation experiments with the expression of either *greA* or *greB* from their native promoters restored unimodal GFP distributions (Fig S2). These results suggested that the impairment in ·NO detoxification in Δ*greA*Δ*greB* originated from bimodal Hmp expression, where a sizable subpopulation of cells did not express Hmp in response to ·NO.

### Expression bimodality extends beyond the *hmp* promoter

**Fig 4:**
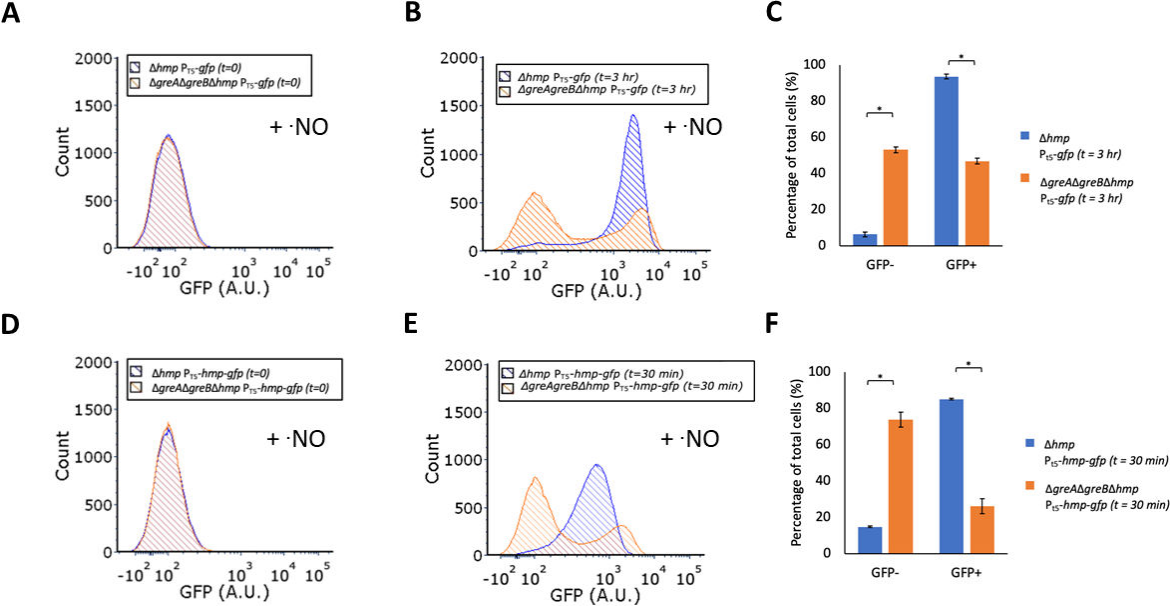
Bimodal expression in Δ*greA*Δ*greB* populations extends to the T5 promoter. Cultures were grown in MOPS minimal media to mid-exponential phase and inoculated into bioreactors at an OD_600_ of 0.05. Immediately after, 250 µM (Panels A–C) or 350 µM (Panels D–F) of DPTA NONOate were added, along with 1 mM IPTG. A slightly higher DPTA NONOate concentration was used for experiments with Hmp-GFP to match peak ·NO concentrations from other experiments. Samples were removed at indicated time points, fixed, and fluorescence was measured by flow cytometry. Panels (A and B) reflect fluorescence distributions for cells containing constructs for T5 promoter expression of GFP at t = 0 (before induction) and t = 3 h (after induction), respectively. Panel C represents the percentage of GFP-positive and GFP-negative cells at t = 3 h. Panels (D and E) reflect fluorescence distributions for cells containing constructs for T5 promoter expression of Hmp-GFP translational fusions at t = 0 (before induction) and t = 30 min (after induction), respectively. Panel F represents the percentage of GFP-positive and GFP-negative cells at t = 30 min. Flow cytometry with Hmp-GFP is depicted at an earlier time point (30 min) such that ·NO would still be present in both cultures. Δ*hmp* (blue) and Δ*greA*Δ*greB*Δ*hmp* (orange). Fluorescent histograms are representative of three or more biological replicates. Bar charts show the means of at least three independent replicates, whereas error bars represent the standard errors of the means. Asterisks indicate statistical significance, at a *P*-value ≤ 0.05, which was assessed by *t*-tests.

We were interested to see whether the bimodality observed from the *hmp* promoter extended to other expression systems. To test this, we expressed GFP from an IPTG-inducible T5 promoter under ·NO stress. We found that expression was again bimodal with this independent expression system (Fig. 4A and B). Approximately 94% of Δ*hmp* synthesized GFP above t = 0 levels in 3 h, whereas significantly fewer Δ*greA*Δ*greB*Δ*hmp* (approximately 47%) generated GFP above initial levels (Fig. 4C). Using an Hmp-GFP translational fusion in place of GFP in the same expression construct, we observed delayed ·NO detoxification (Fig. S3), and bimodal Hmp-GFP expression in Δ*greA*Δ*greB*Δ*hmp*, which was absent from Δ*hmp* (Fig. 4D and E). Notably, within 30 min, 85% of Δ*hmp* had synthesized Hmp-GFP above initial levels, which was significantly higher than that of Δ*greA*Δ*greB*Δ*hmp*, which was 26% of cells (Fig. 4F). Collectively, these data suggested that bimodal expression in Δ*greA*Δ*greB* under ·NO stress was not specific to the *hmp* promoter, but rather it reflected a more general trait of the Gre factor mutant. Moreover, the use of Hmp-GFP provided direct evidence of an association between impaired ·NO detoxification and bimodal expression in Δ*greA*Δ*greB* under ·NO stress.

### Bimodal expression arises from phenotypic heterogeneity

**Fig 5:**
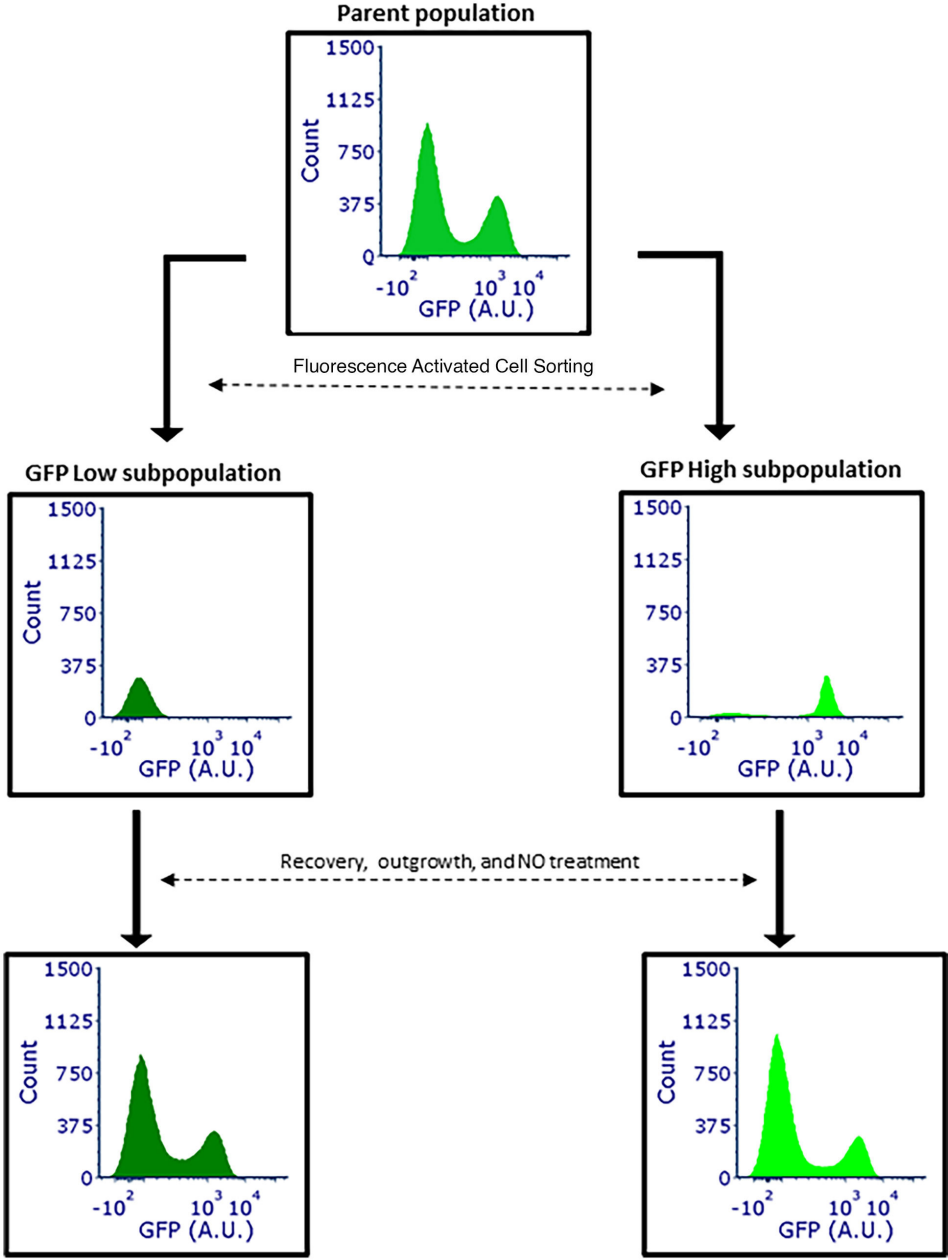
Δ*greA*Δ*greB*Δ*hmp* cells do not retain the memory of GFP expression under prior ·NO stress. Cultures were grown in MOPS minimal media to mid-exponential phase and inoculated into bioreactors at an OD_600_ of 0.05. Immediately after, 250 µM of DPTA NONOate and 1 mM IPTG were added to the bioreactor. Samples were removed at 1 h and passed through a cell sorter to collect 1 million cells from the bottom 10% and top 10% of the fluorescence distribution. Collected cells were recovered in rich media, grown to mid-exponential phase in MOPS minimal media, and dosed with 250 µM DPTA NONOate and 1 mM IPTG. Samples were removed after 1 h, fixed, and fluorescence distributions were measured by flow cytometry. Parent population (medium shade green), GFP low subpopulation (dark shade green), and GFP high subpopulation (light shade green). Images are representative of two biological replicates.

The observation that two populations arise under ·NO stress led us to question whether the phenomenon was heritable, and thus originating from a genetic change, or whether it was transient, and thus phenotypic. To answer this question, we exposed Δ*greA*Δ*greB*Δ*hmp* cultures to ·NO and sorted cells from the top 10% (high) and bottom 10% (low) of the fluorescence distribution after 1 h of ·NO exposure (Fig. 5). Those subpopulations were then cultured and prepared for subsequent assays. Interestingly, cells originating from the high and low subpopulations displayed the same bimodal GFP expression pattern as the parent population after outgrowth, which demonstrated that the expression status of subpopulations was not heritable, but transient. Cell culturability was similar between populations (Fig. S4A and B), and neither subpopulation had a significant difference in terminal cell density or growth dynamics thereafter (Fig. S4C and D). These data showed that bimodal expression in ·NO-stressed Δ*greA*Δ*greB* populations arose from phenotypic heterogeneity, rather than genetic mutation.

### Phenotypic heterogeneity at the level of transcription

**Fig 6:**
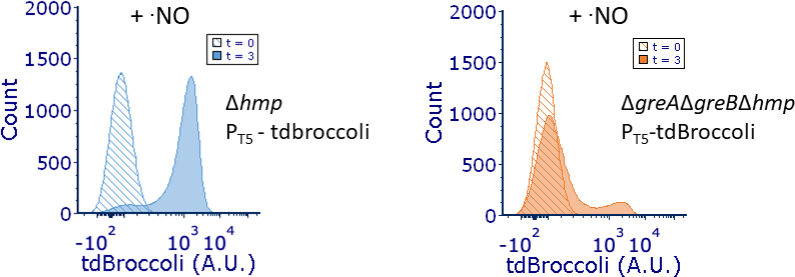
Δ*greA*Δ*greB* displays bimodal transcript production. Cultures of Δ*hmp* and Δ*greA*Δ*greB*Δ*hmp* harboring P_T5_- tdBroccoli were grown in MOPS minimal media to mid-exponential phase and inoculated into a bioreactor at an OD_600_ of 0.05. Immediately after, 250 µM of DPTA NONOate, 1 mM IPTG, and 50 µM DFHBI-1T were added to the bioreactor. Fluorescence distributions at the 0- and 3-h time points are depicted. Δ*hmp* (blue) and Δ*greA*Δ*greB*Δ*hmp* (orange). Images are representative of at least three biological replicates.

**Fig 7:**
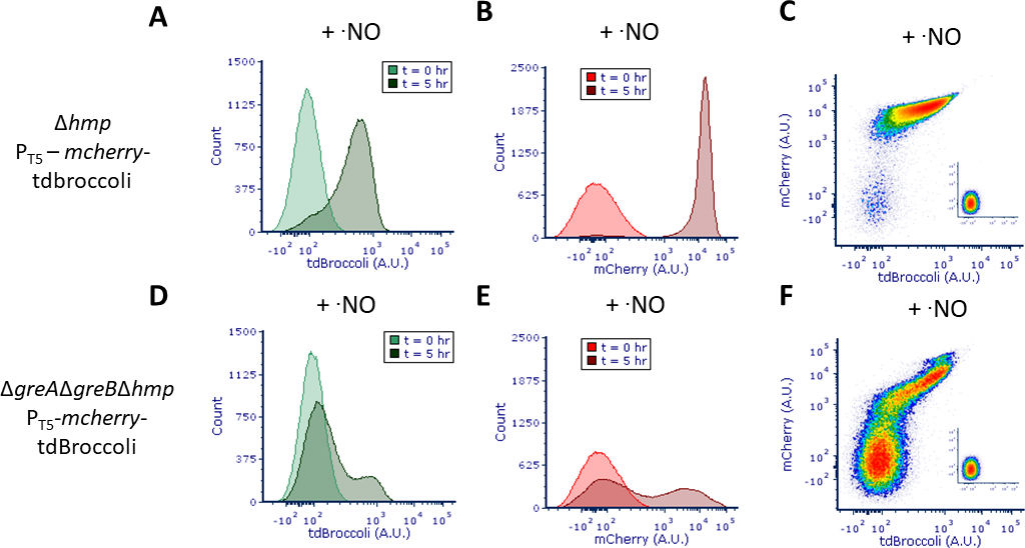
Positive correlation between transcript and protein expression in Δ*greA*Δ*greB* subpopulations. Cultures of Δ*hmp* and Δ*greA*Δ*greB*Δ*hmp* harboring P_T5_-*mcherry*-tdBroccoli were grown in MOPS minimal media to mid-exponential phase and inoculated into a bioreactor at an OD_600_ of 0.05. Immediately after, 250 µM of DPTA NONOate, 2 mM IPTG, and 50 µM DFHBI-1T were added to the bioreactor. After 5 h of treatment, samples were removed and tdBroccoli and mCherry fluorescence were measured concurrently by flow cytometry. Panels (**A**) and (**B**) represent Δ*hmp* tdBroccoli and mCherry distributions, respectively. Panels (**D**) and (**E**) depict Δ*greA*Δ*greB*Δ*hmp* tdBroccoli and mCherry, respectively. Panels (**C**) and (**F**) represent tdBroccoli vs mCherry distributions for Δ*hmp* and Δ*greA*Δ*greB*Δ*hmp,* respectively at t = 5 h, whereas subplots represent t = 0 h. Images are representative of at least three biological replicates.

To further understand this phenomenon, we sought to assess whether the phenotypic heterogeneity we observed was present at the level of transcription or whether it originated from a post-transcriptional process since the outputs we were measuring, Hmp-GFP and GFP, were proteins. To accomplish that, we used an RNA aptamer (tdBroccoli) capable of emitting green fluorescence upon complexing with a cell-permeable, non-fluorescent fluorophore (DFHBI-1T) (Fig. S5A) (43–46). Neither tdBroccoli, nor DFHBI-1T produced appreciable fluorescence on their own (Fig. S5B) (45). We expressed tdBroccoli from an IPTG-inducible promoter in Δ*hmp* and Δ*greA*Δ*greB*Δ*hmp* cultures. In the absence of ·NO, both Δ*hmp* and Δ*greA*Δ*greB*Δ*hmp* displayed unimodal fluorescence distributions (Fig. S5C). However, in the presence of ·NO stress, we found that Δ*greA*Δ*greB*Δ*hmp* cells displayed bimodal fluorescence, suggesting that the phenotypic heterogeneity observed included contributions at the level of transcription (Fig. 6). To demonstrate this directly, we transcriptionally fused mCherry to the 5′ end of tdBroccoli (46, 47). Such an expression system allows direct quantification of both transcriptional and protein output, simultaneously. We found that, under ·NO stress, Δ*hmp* displayed a unimodal expression pattern in both transcript and protein (Fig. 7A and B) with the vast majority of cells increasing transcript and protein levels concurrently (Fig. 7C). On the other hand, Δ*greA*Δ*greB*Δ*hmp* displayed bimodal transcript and protein expression patterns (Fig. 7D and E), where one subpopulation simultaneously contained low transcript and protein abundance, and the other subpopulation concurrently increased transcript and protein expression (Fig. 7F). Collectively, these data suggested that, under ·NO stress, Δ*greA*Δ*greB* cells can enter one of two states, one that is inactive and impaired in transcription, which consequently impacts translation, and a second that produces transcripts comparable to WT, which consequently led to protein output that was comparable to WT.

### Phenotypic heterogeneity is solely attributable to transcription

**Fig 8:**
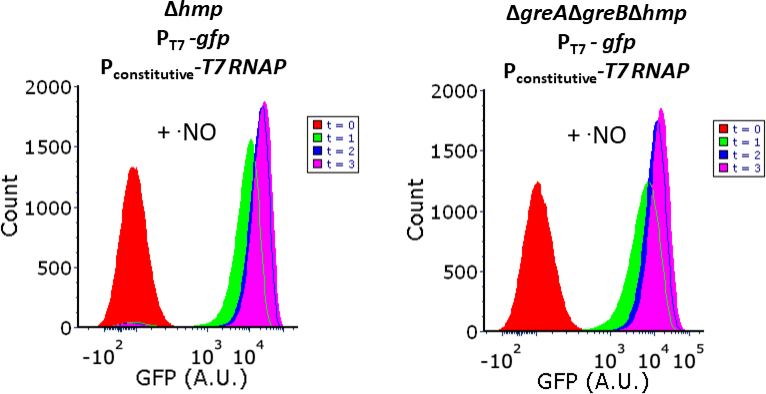
GFP expressed unimodally from orthogonal transcription system in Δ*greA*Δ*greB*. Δ*hmp* and Δ*greA*Δ*greB*Δ*hmp* cultures harboring T7 RNAP under a constitutive promoter (J23114) and P_T7_-*gfp* were grown in MOPS minimal media to mid-exponential phase and inoculated into bioreactors at an OD_600_ of 0.05. Immediately after, 100 µg/mL rifampicin was added to the bioreactor. After 10 min, 250 µM of DPTA NONOate and 1 mM IPTG were added. Samples were removed at t = 0, 1, 2, and 3 h, fixed, and GFP fluorescence distributions were obtained by flow cytometry analysis. Images are representative of at least three biological replicates.

Consistent with the established functions of GreA and GreB acting on RNAP (27, 30, 31), experiments with mCherry-tdBroccoli demonstrated that bimodal transcript levels were a driver of bimodal protein levels. However, data in Fig. 7 did not rule out the possibility that post-transcriptional events were contributing to the phenotypic heterogeneity observed. To assess whether post-transcriptional processes were playing a role, we used the bacteriophage T7 RNA polymerase (T7 RNAP), a non-native, single-unit polymerase, that is evolutionarily distinct from *E. coli* RNAP, and is not known to interact with Gre factors (48, 49). T7 RNAP was expressed from a constitutive *E. coli* promoter so that it would be present as a functional protein during assays, and rifampicin was added to block further transcription from *E. coli* RNAP (including that of T7 RNAP), whereas *gfp* was expressed from an IPTG-inducible T7 promoter, which is not recognized by *E. coli* RNAP (Fig. S6). With this design, transcription of *gfp* under rifampicin treatment would be solely attributable to T7 RNAP, without a contribution from *E. coli* RNAP, and thus post-transcriptional events could be assessed for phenotypic heterogeneity independent of *E. coli* RNAP. Using this system with Δ*hmp* and Δ*greA*Δ*greB*Δ*hmp*, cells were treated with ·NO and rifampicin, and IPTG was added to induce GFP production. We observed a unimodal distribution pattern for both Δ*hmp* and Δ*greA*Δ*greB*Δ*hmp* cultures expressing GFP from the T7 promoter (Fig. 8). Switching to a non-native RNAP expression system eliminated phenotypic heterogeneity, which provided evidence that post-transcriptional mechanisms did not contribute to the phenomenon. Furthermore, it suggested that phenotypic heterogeneity was solely dependent upon transcription performed by *E. coli* RNAP, which is consistent with the primary function of Gre factors as regulators that act through the RNAP secondary channel (27, 30, 31).

### Stress-induced heterogeneity without Gre factors with H_2_O_2_

**Fig 9:**
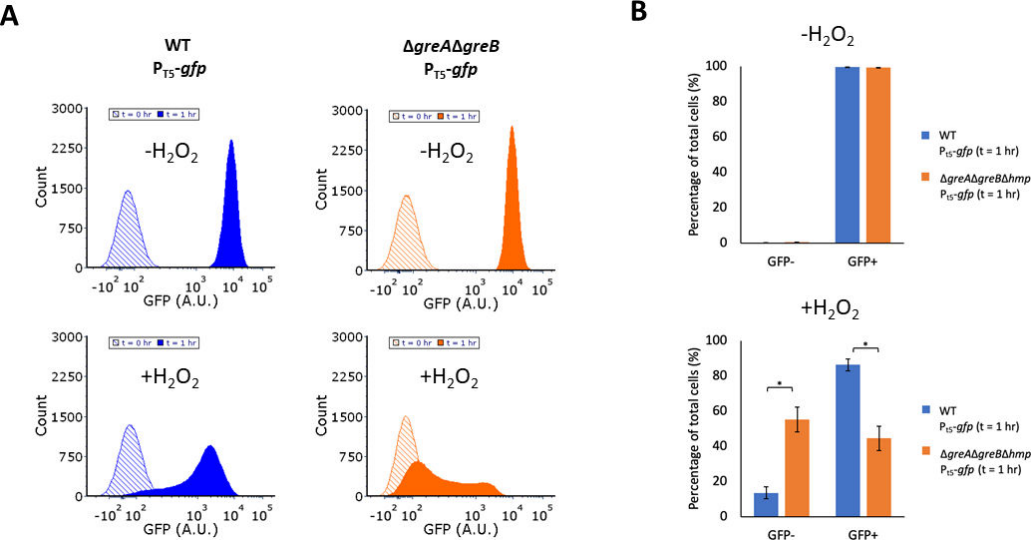
Bimodal expression without Gre factors occurs under H_2_O_2_ stress. Cultures were grown in M9 minimal media to mid-exponential phase and inoculated into a bioreactor containing 75 µM H_2_O_2_ (+H_2_O_2_ condition) or the same volume of autoclaved Milli-Q water (-H_2_O_2_ condition). Immediately after 1 mM IPTG was added. (**A**) Samples were removed at 0 and 1 h, fixed, and GFP distributions were measured by flow cytometry. (**B**) Bar charts show the percentage of GFP-positive and GFP-negative cells at t = 1 h with and without (±) H_2_O_2_ treatment. WT (blue), Δ*greA*Δ*greB* (orange). Images are representative of at least three biological replicates. Bar charts show the means of at least three independent replicates, whereas error bars represent the standard errors of the means. Asterisks indicate statistical significance, at a *P*-value ≤ 0.05, which was assessed by *t*-tests.

Given the results with ·NO, we sought to assess the generality of stress-induced phenotypic heterogeneity in Δ*greA*Δ*greB* populations. To accomplish that we elected to test H_2_O_2_, which is another toxic metabolite present within phagosomes that is small, uncharged, catalytically detoxified by enzymes, and potentially genotoxic depending on its concentration (8, 42, 50). Specifically, we induced expression of GFP from an IPTG-inducible P_T5_ promoter in WT and Δ*greA*Δ*greB* in the presence and absence of H_2_O_2_ and monitored fluorescence with flow cytometry. We found unimodal expression for both WT and Δ*greA*Δ*greB* in the absence of H_2_O_2_, whereas exposure to 75 µM H_2_O_2_ led to bimodal GFP expression in Δ*greA*Δ*greB* populations and unimodal expression in WT (Fig. 9). While 86% of WT cells induced GFP expression above background levels in the presence of H_2_O_2_, only 45% of Δ*greA*Δ*greB* cells induced GFP expression, which was significantly less than that of WT (Fig. 9). We note that 25 and 50 µM doses were also tested, and while bimodality in 50 µM treatments of Δ*greA*Δ*greB* begins to emerge at 1 h, the 25 µM treatment remained unimodal through 1 h (Fig. S7A). Previous work in similar conditions established that 25 µM treatments of H_2_O_2_ are cleared by 1 h (50), and thus these data would suggest that the duration of time under stress is impactful to whether bimodal distributions have time to develop in Δ*greA*Δ*greB* populations under stress. Collectively, these data show that stress-induced phenotypic heterogeneity of Δ*greA*Δ*greB* populations is not confined to ·NO, but extends to another prevalent toxic metabolite, H_2_O_2_.

### Cheating occurs in Δ*greA*Δ*greB* populations under ·NO stress

**Fig 10:**
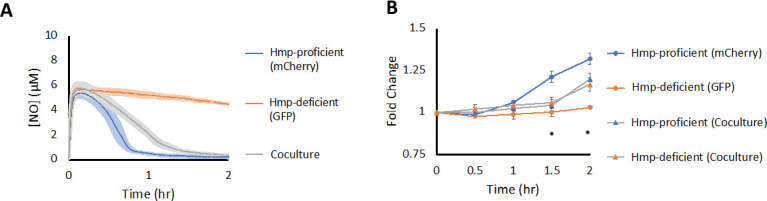
Cheating occurs in ·NO-treated cocultures of Hmp-proficient and Hmp-deficient strains. Cultures were grown in MOPS minimal media to mid-exponential phase and inoculated into a bioreactor at an OD_600_ of 0.025 either as a monoculture of Hmp-proficient cells (blue), a monoculture of Hmp-deficient cells (orange), or as a 1:1 coculture of both strains (gray). Immediately after, 250 µM of DPTA NONOate was added to the bioreactor. (**A**) ·NO concentrations were continuously monitored in the bioreactor. Solid lines represent the means of at least three independent replicates, whereas light shading represents the standard errors of the means. (**B**) Samples were removed to measure OD_600_ at indicated time points. For co-cultures, samples were fixed and the OD_600_ was scaled by the proportion of GFP- and mCherry-positive cells measured by flow cytometry. Colored circles and triangles represent the means of at least three replicates, whereas error bars represent the standard errors of the means. Asterisks indicate statistical significance, at a *P*-value ≤ 0.05, which was assessed by one-way ANOVA and post hoc Tukey HSD tests. At 1.5 h, Hmp-proficient (mCherry) samples are significantly different from all other samples; at 2 h, Hmp-deficient (GFP) samples are significantly different from all samples, and co-culture strains are not significantly different at any time points.

Given the results described above, we considered the potential ramifications of phenotypic heterogeneity under toxic metabolite stress. Figure 1 showed that heterogeneous populations (Δ*greA*Δ*greB*) resumed growth after ·NO stress at much later times than WT, which produced unimodal populations. Previous work established that Hmp detoxifies approximately 99.8% of intracellular ·NO under similar conditions and that after a short induction period cellular ·NO detoxification vastly exceeds abiotic loss of ·NO (e.g., autooxidation, gas phase transport) (40). In consideration of this knowledge and the fact that ·NO is a small uncharged molecule that diffuses rapidly, we reasoned that the burden of ·NO detoxification in heterogeneous populations would fall upon the Hmp-expressing subpopulation, whereas the non-responsive subpopulation would not need to commit resources to deal with ·NO but could benefit from the other subpopulation clearing ·NO from the environment. Such a scenario would constitute cheating by the non-responsive subpopulation, which is not present in WT cultures. To examine the plausibility of this scenario, we performed experiments with monocultures and cocultures of Hmp-proficient and Hmp-deficient strains. To enable the differentiation of strains in coculture, we labeled them with different fluorophores. Specifically, the Hmp-proficient strain constitutively expressed mCherry, whereas the Hmp-deficient strain constitutively expressed GFP. Consistent with previous findings, monocultures of the Hmp-proficient strain cleared ·NO and resumed growth significantly faster than monocultures of Hmp-deficient strains (Fig. 10). Furthermore, we found that 50/50 cocultures of Hmp-proficient and Hmp-deficient strains cleared ·NO slower than the Hmp-proficient monoculture (Fig. 10A), and when growth was monitored, the fold-change in cell number for the Hmp-proficient and Hmp-deficient strains in coculture were comparable and both lower than the Hmp-proficient monoculture (Fig. 10B). These data suggested that phenotypic heterogeneity under ·NO stress can produce scenarios where one subpopulation cheats off of another subpopulation.

**Fig 11:**
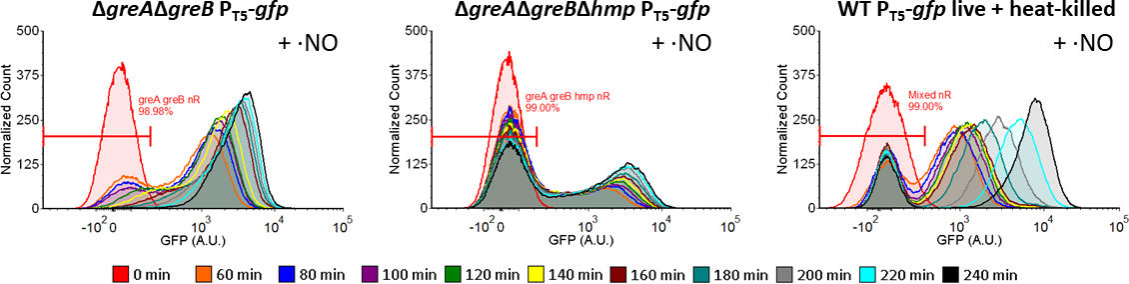
Flow cytometry histograms during ·NO treatment show cheating in Δ*greA*Δ*greB* cultures. Cultures of Δ*greA*Δ*greB*, Δ*greA*Δ*greB*Δ*hmp*, and WT harboring P_T5_-*gfp* were grown in MOPS minimal media to OD_600_ = 0.2. Δ*greA*Δ*greB* P_T5_-*gfp* and Δ*greA*Δ*greB*Δ*hmp* P_T5_-*gfp* were inoculated into bioreactors to OD_600_ of 0.05. Live and heat-killed WT P_T5_-*gfp* were inoculated as a mixed population into bioreactors to OD_600_ = 0.03 at a ratio of ~2:1 (live:heat-killed). Immediately after inoculation, cultures were treated with 300 µM of DPTA NONOate, and IPTG was added 30 seconds later. Samples were collected before the addition of DPTA NONOate (t = 0 min) and then every 20 min beginning 1 h after treatment. Samples were fixed, fluorescent counting particles were added, and flow cytometry was performed to measure GFP fluorescence. Shown are representative histograms of the GFP fluorescence distributions of cell events, scaled to a total of 50,000 cell events per sample. Gates used to identify non-responding subpopulations are depicted. Δ*greA*Δ*greB* (left), Δ*greA*Δ*greB*Δ*hmp* (middle), live and heat-killed WT coculture (right). Images are representative of at least three biological replicates.

**Fig 12:**
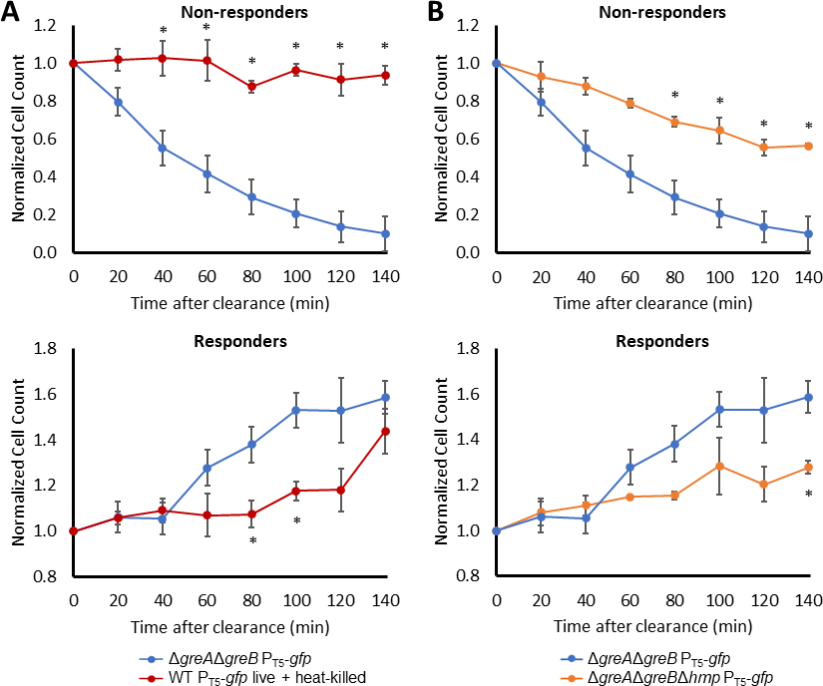
Non-responding subpopulations of Δ*greA*Δ*greB* rapidly increase GFP expression after ·NO clearance compared to controls. Sample preparation and assay details are identical to those of Fig. 11. (**A**) Cell counts plotted here begin at the last time point before ·NO was cleared (t = 0 min) and are normalized to t = 0 min. (**B**) Cell counts plotted here begin at the last time point before ·NO was cleared from Δ*greA*Δ*greB* cultures (t = 0 min), which was done because Δ*greA*Δ*greB*Δ*hmp* cultures do not clear ·NO, and are normalized to t = 0 min. Data points show the means of at least three independent replicates, whereas error bars represent the standard errors of the means. Asterisks indicate statistical significance between Δ*greA*Δ*greB* P_T5_-*gfp* and associated control at each time point, at a *P*-value ≤ 0.05, which was assessed by *t*-tests. Absolute cell counts in cells per mL corresponding to the normalized cell counts depicted here are provided in Fig. S8J through O.

Inspired by results from the Hmp-proficient and Hmp-deficient model system, we sought to directly determine whether cheating occurred in Δ*greA*Δ*greB* populations under ·NO stress. To accomplish that, we measured the absolute abundances of responsive and non-responsive cells in Δ*greA*Δ*greB* populations after ·NO clearance with flow cytometry and counting beads. Responsive cells were defined as those that expressed GFP to levels higher than that of 99% of cells prior to IPTG induction, and non-responsive cells were those that failed to do so. We hypothesized that if cheating occurred the absolute abundance of non-responsive cells would decline after ·NO had been cleared by the responsive subpopulation, whereas if cheating did not occur the absolute abundance of non-responsive cells would remain the same. As depicted in Fig. 11, prior to ·NO clearance the Δ*greA*Δ*greB* population was bimodal, and after ·NO clearance (120 min and later) the non-responsive subpopulation began to uniformly increase GFP expression. This shift was not observed in a control system comprised of responsive (WT) and permanently non-responsive cells (heat-killed WT), which could detoxify ·NO at a comparable rate as Δ*greA*Δ*greB* (Fig. S8C), or in the absence of ·NO detoxification (Δ*greA*Δ*greB*Δ*hmp*) (Fig. 11). When cell numbers were quantified with counting beads and flow cytometry, we observed that the absolute abundance of non-responsive cells in Δ*greA*Δ*greB* cultures decreased significantly after ·NO clearance to levels that were ~90% lower, whereas the responsive cells in those populations significantly increased in absolute abundance during the same time period (Fig. 12; Fig. S8D and E). With the control system with a permanently non-responsive subpopulation, minimal changes in the absolute abundances of non-responsive cells were observed after ·NO clearance, whereas the responsive subpopulation significantly increased in number (Fig. 12A; Fig. S8F and G). In the absence of ·NO detoxification (Δ*greA*Δ*greB*Δ*hmp*), modest changes in the absolute abundances of the non-responsive and responsive subpopulations were observed over the same timepoints (Fig. 12B; Fig. S8H and I), and those magnitudes were significantly less than those of Δ*greA*Δ*greB* populations. Importantly, these data are from direct measurements of absolute cell counts of non-responsive and responsive cells, and neither were inferred from changes in the other. The y-axes are cell counts normalized to time 0 of the same time series experiment and the absolute cell/mL data are provided in Fig. S8J through O. These data demonstrate that non-responsive subpopulations of Δ*greA*Δ*greB* cheat off of their responsive counterparts because once ·NO has been cleared by responsive cells, the vast majority of cells in non-responsive subpopulations begin to synthesize protein.

**Fig 13:**
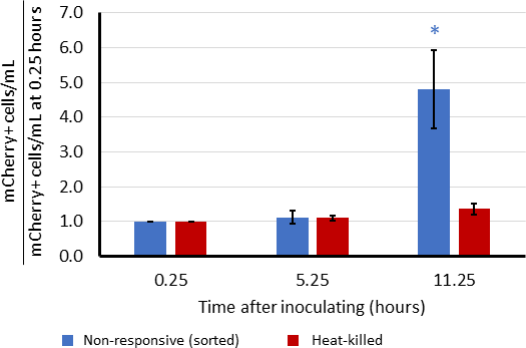
Non-responding Δ*greA*Δ*greB* cells grow after ·NO clearance. Cultures of Δ*greA*Δ*greB* that express mCherry constitutively and carry an IPTG-inducible GFP on pUA66 were grown in MOPS minimal media to OD_600_ = 0.2 and inoculated into bioreactors to OD_600_ of 0.02. Immediately after inoculation, cultures were treated with 300 µM of DPTA NONOate and IPTG was added 30 seconds later. WT harboring an empty pUA66 were grown in MOPS minimal media to OD_600_ = 0.2, inoculated into bioreactors to OD_600_ of 0.01, and treated with 300 µM of DPTA NONOate. WT bioreactors were initiated 30 min after Δ*greA*Δ*greB* bioreactors. Δ*greA*Δ*greB* cultures were collected and non-responsive (low GFP) cells were sorted using fluorescence-activated cell sorting (FACS). Sorted non-responsive Δ*greA*Δ*greB* cells were inoculated into WT bioreactors when [·NO] was approximately 2 µM (Fig. S8P). Heat-killed cultures of the same Δ*greA*Δ*greB* strain were used as controls for cells that cannot resume growth, which were inoculated into separate WT bioreactors. Samples were fixed at indicated times and mCherry-positive cells were counted by flow cytometry with counting beads. Data points show the means of at least three independent replicates, whereas error bars represent the standard errors of the means. Asterisks indicate statistical significance between mCherry-positive cells at indicated times compared to the initial timepoint at a *P*-value ≤ 0.05, which was assessed by *t*-tests.

As an additional line of evidence for cheating within Δ*greA*Δ*greB* populations under ·NO stress, we sought to fluorescently label Δ*greA*Δ*greB*, sort out non-responsive cells from ·NO-stressed Δ*greA*Δ*greB* cultures, inoculate those non-responsive cells into a wild-type (unlabeled) population, and demonstrate that after ·NO had been detoxified the non-responsive Δ*greA*Δ*greB* cells that had been inoculated then resumed growth. To accomplish this, we constructed a Δ*greA*Δ*greB* strain that expressed mCherry constitutively from a P_N25_ promoter on the chromosome (DS015) and expressed GFP from an IPTG-inducible T5 promoter on pUA66 (pSA21). The unlabeled population was WT carrying an empty pUA66. Non-responsive cells were sorted from an ·NO-stressed culture of DS015 carrying pSA21, which had been induced with IPTG immediately following ·NO treatment. The non-responsive sorted cells were then inoculated into a bioreactor containing WT with pUA66 that had been treated with ·NO approximately 1 h beforehand (Fig. S8P). mCherry events were then measured before, approximately when, and after ·NO was eliminated from the bioreactor. As a control, heat-killed DS015 carrying pSA21 was used in place of the non-responsive sorted population. Notably, these data were also from direct measurements of absolute cell counts using counting beads. As depicted in Fig. 13, the abundance of mCherry events in the heat-killed control did not vary from the initial time point, whereas the sorted non-responsive cells significantly increased in abundance only after ·NO had been eliminated from the bioreactor. These data also support our conclusion that non-responsive subpopulations of Δ*greA*Δ*greB* cheat off of their ·NO-detoxifying neighbors because once their neighbors remove ·NO, the non-responsive subpopulations begin to replicate.

## DISCUSSION

·NO is a broad-spectrum antimicrobial and a sizeable number of bacteria rely on ·NO detoxification systems to impart their virulence (8, 10, 11, 51). As such, an increased understanding of bacterial ·NO defense networks could identify novel therapeutic targets for the treatment of bacterial infections (8, 9, 18, 40, 41, 52–55). Recently, the global transcriptional regulator, DksA, has been revealed to play an important role in both *S. Typhimurium* ·NO defenses and virulence, as well as ·NO detoxification in *E. coli* (15, 16, 18). DksA is a member of a larger class of proteins that exert their function by binding to the secondary channel of RNAP (23, 27). Among these proteins are Gre factors, that share structural homology with DksA (21, 22, 24). Given the importance of DksA to the ·NO stress response of *E. coli*, we hypothesized that other regulators of the secondary channel of RNAP may influence ·NO detoxification.

Acting to relieve transcriptional stalling, Gre factors are a widely conserved class of proteins across the prokaryotic kingdom that interact with the secondary channel of RNAP (30, 31, 33). Transcriptional stalling is a ubiquitous event, thought to occur during a variety of biochemical activities, including initiation of transcription, recognition of transcriptional errors, collisions with DNA-bound protein complexes, DNA damage, and nucleotide deprivation (21, 32, 56, 57). As such, Gre factors play a crucial role in transcriptional homeostasis, by aiding in RNAP promoter escape, rescuing arrested RNAP complexes, and even serving a proofreading role by cleaving mis-incorporated nucleotides from nascent transcripts (21, 33). Gre factors have also been implicated in the survival of numerous microbes in harsh and restrictive environments. For example, loss of GreA in *M. tuberculosis* and *Mycobacterium smegmatis* led to a significant reduction in survival within macrophages (37), whereas with *Salmonella*, loss of both Gre factors significantly reduced epithelial invasion (35). With mouse experiments, Gre factors were found to be important for *Salmonella* pathogenicity in an NADPH oxidase-dependent manner, which suggested a critical role for them in oxidative stress responses (39). Interestingly, RNAP occupancy on the *Salmonella* genome was found to be more perturbed by loss of *greA* and *greB* during infection than growth in broth (58). In *Francisella tularenensis,* the inactivation of GreA led to reduced invasion and growth within macrophages, as well as reduced survival in mouse models (36). For numerous other bacteria, Gre factors have been implicated as stress proteins, upregulated under harsh environmental conditions that include acid stress, oxidative stress, heat shock, hypoxia, and salt stress (59–63). Indeed, when synthetically over-expressed, GreA increased the survival of *E. coli* cultures that were challenged with heat shock (48°C) or oxidative stress (5 mM H_2_O_2_) (64). Furthermore, an analysis of Gre-regulated transcriptional pauses revealed a vast regulatory network enriched in transcription regulators, which suggested a broad role for Gre factors in bacterial physiology (65).

The evidence suggesting that Gre factors play an important role in intracellular pathogen survival, coupled with their similarities to DksA, inspired us to explore whether such factors play a role in modulating the ·NO stress response of *E. coli*. We found that individual deletion of either *greA* or *greB* had no impact; however, combined loss of both genes significantly impaired ·NO detoxification (Fig. 1). Cells lacking *greA* and *greB* did not display a general growth defect in the absence of ·NO stress (Fig. 2), and we confirmed that the difference in ·NO detoxification observed could be attributed to synthesis of and enzymatic detoxification by Hmp (Fig S1G and H). We demonstrated that Δ*greA*Δ*greB* populations exhibited bimodal expression under ·NO stress (Fig. 3 and 4), where one subpopulation had trouble synthesizing transcripts, whereas the other produced transcripts at levels comparable to WT (Fig. 7). Notably, bimodal expression in ·NO-stressed Δ*greA*Δ*greB* was observed for both the *hmp* and T5 promoters, which shows that the phenomenon was not confined to just the *hmp* promoter. Sorting experiments confirmed that it was phenotypic heterogeneity, rather than a heritable genetic change (Fig. 5), and further experiments with an independent RNAP, that of the bacteriophage T7, revealed that the phenotypic heterogeneity depended on bacterial RNAP (Fig. 8). However, we did not delineate the exact mechanism of how the loss of Gre factors led to stress-induced phenotypic heterogeneity by RNAP in this study. Given the potential of ·NO autoxidation products to damage DNA and stall RNAP, we postulate that differential damage to or repair of DNA can produce subpopulations with arrested RNAPs in the absence of Gre factors. Alternatively, given the regulatory role of Gre factors on gene expression, it is possible that downstream effects of Gre factor loss prime *E. coli* for phenotypic heterogeneity upon toxic metabolite exposure (21, 22, 24, 27, 65). Given the ubiquity of Gre factors in prokaryotes and the inherently stressful lives of microbes, unraveling the molecular mechanism of how loss or inactivity of Gre factors leads to stress-induced phenotypic heterogeneity will be a fertile area for future study. Furthermore, previous work illustrated a role for Gre factors in limiting the epigenetic switching of the *lac* gene network under unstressed growing conditions, which suggests that Gre factors play a role in preventing heterogeneity under a broad swath of environments (66).

Interestingly, phenotypic heterogeneity is often hypothesized to promote bet-hedging when pathogens encounter variable environments (67–69). For example, enteric pathogens such as *E. coli* and *Salmonella* experience drastically different environments while invading the gastrointestinal tract (70). The esophagus, stomach, intestines, and subepithelial tissues are unique environments with differing stress conditions, including shifts from acidic to basic conditions, high to low oxygen levels, and the presence or absence of important nutrients such as iron (70). Heterogeneity is thought to foster adaptation of populations as a whole at the cost of reduced fitness for specific subpopulations (70, 71). However, a potential caveat for the benefits of heterogeneity is cheating in the presence of community variables. ·NO and H_2_O_2_ are highly diffusible toxic metabolites that act effectively as community poisons until their concentrations fall below inhibitory levels. Due to their high diffusivity, cells cannot propagate until the environment has been cleared of ·NO or H_2_O_2_, and thus proficient detoxifiers will commit resources to deal with more than their fair share of poison if neighbors are deficient at detoxification. Such a scenario ultimately delays the elimination of the community insult, leads to prolonged periods of stress, and negatively impacts the entire population since the fittest in those conditions do not increase in abundance compared to those that are the least fit. Indeed, those outcomes were what we observed while studying cheating in Δ*greA*Δ*greB* populations under ·NO stress. For example, Δ*greA*Δ*greB* clears ·NO slower than the wild type, which renders it under stressful conditions longer and delays its growth resumption (Fig. 1). Once ·NO has been cleared, non-responsive cells of Δ*greA*Δ*greB* reactivate and begin to synthesize protein and grow, which was not observed in a control with permanently non-responsive cells (Fig. 11 to 13). In addition, using mono- and co-cultures of Hmp-sufficient and Hmp-deficient strains, we observed delayed ·NO clearance, prolonged ·NO stress, and resumption of growth of the non-responsive subpopulation (Hmp-deficient) that matched that of the responsive subpopulation (Hmp-proficient) and was less than that of a completely responsive population (Fig. 10). Collectively, these series of experiments demonstrate that the non-responsive subpopulations of ·NO-stressed Δ*greA*Δ*greB* cultures cheat off of their responsive counterparts. Given the pervasiveness of toxic metabolites in natural and anthropogenic settings (4, 72, 73), the role of Gre factors in modulating cheating behavior and competition between microbes is a rich area for future research.

In conclusion, Gre factors play vital regulatory roles in transcriptional homeostasis, and their importance to the pathogenicity of numerous microbes continues to be uncovered (34–38). The data presented here add to the growing understanding of Gre factors and their role in bacterial stress physiology. Moreover, results from this work suggest that inhibitors of Gre factors could decrease the virulence of pathogens that rely on the detoxification of toxic metabolites to foster their infectivity. Such agents would work independently of current antibiotics and since the loss of Gre factors does not lead to growth inhibition under normal conditions, it would be projected that the timescale for resistance development would be longer than conventional treatments (74, 75).

## MATERIALS AND METHODS

### Bacterial strains

All strains were derived from *E. coli* K12 MG1655 (WT). Deletion mutants were generated by P1 bacteriophage-mediated transduction from the associated mutant in the Keio collection, which has been described previously, with the exception of *lacI::kanR*, *araBAD::P_T5_-mcherry-kanR, araBAD::P_T5_-gfp-kanR,* and *araBAD::P_N25_-mcherry-gentR,* that were introduced using the lambda red system (76, 77). Following P1 transduction, strains were cured of the chromosomally inserted kanamycin resistance marker using a pCP20 plasmid, which expresses FLP recombinase (77). For double and triple deletion mutants, consecutive rounds of P1 transduction and curing were performed. For a list of strains used in this study, refer to Table S1. All deletions were confirmed by PCR. Refer to Table S2A and B for a list of primers used in mutant construction and verification.

### Chemicals and growth media

The growth media used in this study were Luria Bertani (LB) broth, and MOPS minimal media and M9 minimal media, which were both supplemented with 10 mM glucose as the sole carbon source. LB broth was made by combining LB powder (40% tryptone, 20% yeast extract, 40% NaCl per gram of solid) with Milli-Q water (18.2 MΩ • cm at 25°C) and sterilizing the solution by autoclaving. MOPS and M9 minimal media were sterilized by filtration. The ·NO donor used, (Z)−1-[N-(3-aminopropyl)-N-(3-ammoniopropyl)amino]diazen-1-ium-1,2-diolate (DPTA NONOate) (Cayman Chemical), was dissolved in 10 mM NaOH to a concentration of 72 mM and stored on ice. Kanamycin, ampicillin, gentamicin, and isopropyl ß-D-1-thiogalactopyranoside (IPTG) were dissolved in autoclaved Milli-Q water at concentrations of 50 mg/mL, 100 mg/mL, 30 mg/mL, and 0.238 g/mL, respectively, sterile-filtered and stored at 4°C or -20°C prior to use. DFHBI-1T was resuspended in DMSO at a concentration of 5 mg/mL. For ·NO probe calibrations, a solution of SNAP (S-Nitroso-N-Acetyl-D,L-Penicillamine) and ethylenediaminetetraacetic acid (EDTA) was prepared by reconstituting 5.6 mg SNAP and 5 mg EDTA in 25 mL autoclaved Milli-Q water. A 0.1 M CuCl_2_ solution was prepared by dissolving 8.5 g CuCl_2_·2H_2_O in 500 mL of Milli-Q water. For H_2_O_2_ treatment assays, an H_2_O_2_ solution (35% wt solution in H_2_O—Fisher Scientific) was dissolved in autoclaved Milli-Q water at a concentration of 25, 50, or 75 mM.

### Plasmids

For a list of plasmids used in this study, refer to Table S1. All plasmids were derived from either a low-copy (pUA66) or a high-copy plasmid (pQE80), and the parental plasmid for each construct is specified in Table S1. pJR05, pSA21, pXW02, pXW09, and pTOX66 were obtained from previous works. pDS01 and pDS02 were constructed by restriction digestion with BamHI and SbfI followed by ligation with a Quick Ligation Kit (NEB). All other plasmids except pDS09 were constructed using a HiFi DNA assembly kit (NEB). All plasmids were confirmed by Sanger sequencing (Genewiz). pDS09 was constructed using Gibson assembly (NEB) and confirmed by PCR. Refer to Table S2C and D for a list of primers and double-stranded DNA sequences used for plasmid construction.

### ·NO measurements

·NO concentrations in bioreactors were continuously measured using an ·NO-sensing electrode (World Precision Instruments). Daily calibrations were performed by submerging the probe in a solution of 100 mM CuCl_2_ and adding increasing doses of SNAP. The raw signals generated from the probe, in units of picoamps, were converted to units of ·NO concentration (in μM) using a scaling factor of 0.457 ·NO per molecule of SNAP, which was determined previously (18).

### H_2_O_2_ measurements

H_2_O_2_ concentrations were measured using Amplex Red hydrogen peroxide/peroxidase kits (Life Technologies) according to the manufacturer’s instructions. A standard curve with concentrations of 0, 1, 2.5, 5, and 10 µM H_2_O_2_ was constructed. Samples were diluted to a concentration within 10 µM to convert fluorescence values to H_2_O_2_ concentrations.

### Absorbance and fluorescence measurements

Cell density was measured during experiments by sampling 300 µL of cultures from bioreactors or flasks, and measuring the absorbance at 600 nm using a 96-well clear, flat bottom plate (Corning) and a Synergy H1 Hybrid Microplate Reader. Fluorescence measurements were performed on an LSRII flow cytometer (BD Bioscience). For GFP and tdBroccoli, an excitation wavelength of 488 nm and an emission bandpass filter of 525/50 were used. For mCherry, the excitation wavelength and emission bandpass filter were set to 561 and 610/20, respectively. For each sample, 50,000 cellular events were recorded. For calculating the percentages of GFP-positive and -negative cells, gates were set on t = 0 distributions to include 99% of events as GFP negative (prior to ·NO treatment or the addition of IPTG). Cells above this threshold were considered GFP-positive.

### ·NO and H_2_O_2_ consumption assays

*E. coli* were taken from a −80°C frozen stock, inoculated into 1 mL of LB media, and grown for 6 h in an incubator at 37°C and 250 RPM. After 6 h, 10 µL of culture was transferred to 1 mL of MOPS media and incubated for 16 h at 37°C and 250 RPM. After 16 h, OD_600_ were measured and the corresponding volumes of culture were transferred into 20 mL MOPS media in 250 mL baffled flasks to achieve OD_600_ of 0.01. Flasks were incubated at 37°C and 250 RPM until cultures reached the mid-exponential phase (OD_600_ = 0.2). Then, 8 mL of culture were transferred to eight prewarmed (37°C) microcentrifuge tubes and spun at 15,000 RPM for 3 min. Nine hundred and eighty microliters of supernatant was removed from each tube and cell pellets were resuspended in 1 mL of pre-warmed MOPS media.

For ·NO consumption assays, concentrated cultures were used to inoculate bioreactors containing 10 mL MOPS media to an OD_600_ of 0.05. Immediately after inoculation, DPTA NONOate was added to an initial concentration of 250 µM. For assays involving Hmp-GFP translational fusions, the concentration of DPTA NONOate was increased to 350 µM to achieve similar ·NO concentration profiles as WT cultures. Throughout assays, ·NO concentrations were continuously monitored. Cell density measurements were taken by extracting 300 µL from bioreactors at indicated time points and measuring the OD_600_.

For H_2_O_2_ consumption assays, M9 growth media was used instead of MOPS minimal media, as MOPS had previously been shown to interfere with Amplex Red H_2_O_2_ quantification (78). Immediately before inoculation, H_2_O_2_ was added to reactors to the desired concentrations (25, 50, or 75 µM). Afterward, concentrated cultures were used to inoculate bioreactors to an OD_600_ of 0.01. At each time point, 300 µL of culture was removed and sterile-filtered using a 0.22-µm syringe filter (Millex). Samples for initial time points (t = 0) were removed prior to inoculation with cells. All samples were stored on ice prior to quantification of [H_2_O_2_].

In experiments involving strains harboring plasmids, the appropriate concentrations of antibiotic (50 µg/mL kanamycin or 100 µg/mL ampicillin) were included in growth media at every step.

### Cell survival assay

Cells were prepared following the same procedure as ·NO and H_2_O_2_ consumption assays. After inoculating cells into the bioreactor, 300 µL samples were removed to measure the initial OD_600_, and 10 µL of the 300 µL sample was serially diluted in PBS and plated onto LB-agar. Immediately after, DPTA NONOate was added to the bioreactor at a concentration of 250 µM. For subsequent time points, the same procedure was used to plate cells. Plates were incubated at 37°C for 16 h after which the number of colonies were counted and converted to CFU/mL.

### Fluorescent reporter assays

Cells were prepared following the same procedure as ·NO and H_2_O_2_ consumption assays. For assays involving H_2_O_2_, 25, 50, or 75 µM of H_2_O_2_ was added prior to cells. After inoculating cells into the bioreactor, 300 µL of samples was removed to measure the initial OD_600_ and a second 300 µL of sample was removed and centrifuged at 15,000 RPM for 3 min. After centrifugation, 250 µL of supernatant was removed and pellets were resuspended in 250 µL of 4% paraformaldehyde in PBS (4% PFA). Samples were kept on ice for 30 min, after which they were centrifuged for 3 min at 15,000 RPM, 250 µL of supernatant were removed, the pellet was resuspended in 550 µL PBS, and then stored at 4°C until flow cytometry. After the removal of initial samples (t = 0), for ·NO stress assays, 250 µM DPTA NONOate was immediately added to the bioreactor. For cells harboring IPTG-inducible plasmids, 1 mM IPTG was also added to the bioreactor at that time. For subsequent time points, 300 µL of sample was extracted from bioreactors, and the same procedure was used to prepare samples for flow cytometry. Samples were analyzed on an LSRII flow cytometer (BD Biosciences).

RNA aptamer-based assays followed the same procedure with a few modifications. DFHBI-1T was added to the bioreactor at t = 0 to a final concentration of 50 µM. Samples were not fixed in 4% PFA but instead centrifuged at 15,000 RPM for 1 min, 250 µL of supernatant was removed, pellets were resuspended in ice-cold PBS containing 50 µM DFHBI-1T, and stored on ice prior to flow cytometry. Samples were analyzed on the same day, immediately after collection of the terminal timepoint. For mCherry-tdBroccoli (Fig. 7), 2 mM IPTG was introduced (instead of 1 mM) and terminal samples were removed at t = 5 h (instead of 3 h), to increase fluorescence output and allow the signal to further develop.

For Fig. 2B; Fig. S1F, experiments were performed in 250 mL baffled flasks as opposed to bioreactors. Cultures were inoculated at an OD_600_ of 0.01, and after 2 h of incubation at 37°C and 250 RPM, 300 µL of samples was removed and prepared for flow cytometry following the procedure outlined above. Immediately after, 1 mM of IPTG was added to the flasks. Samples were removed every hour for 3 h and prepared for flow cytometry.

### Cell sorting assay to assess the heritability of a phenotype

Cells were prepared following the same procedure as ·NO and H_2_O_2_ consumption assays. At t = 0, 250 µM DPTA NONOate and 1 mM IPTG were immediately added to the bioreactor. After 1 h, 1 mL of samples was removed and spun at 15,000 RPM for 3 min, 950 µL of supernatant was removed, and pellets were resuspended in 1,950 µL ice-cold PBS and stored on ice. Samples were sorted on a fluorescence-activated cell sorting (FACS) ARIA fusion (BD Biosciences) in which 1 million high-GFP cells (top 10th percentile), 1 million low-GFP cells (bottom 10th percentile), and 1 million cells from the total distribution were collected in microcentrifuge tubes and stored on ice. Collected samples were centrifuged at 15,000 RPM for 3 min, 950 µL of supernatant were removed, and pellets were resuspended in 950 µL of LB containing 50 µg/mL kanamycin. Samples were transferred to test tubes and incubated at 37°C and 250 RPM for 12 h. Afterward, 500 µL of sample was mixed with 500 µL of 50% glycerol in cryotubes and stored at −80°C. For culturability measurements after cell sorting (Fig. S4A and B), 500,000 cells were collected instead, and sorted samples were immediately diluted in PBS and plated onto LB-agar containing 50 µg/mL kanamycin.

### Hmp-proficient and Hmp-deficient coculture experiments

Cultures of Hmp-proficient (mCherry) and Hmp-deficient (GFP) cells were prepared concurrently using the same procedure as ·NO and H_2_O_2_ consumption assays. Both strains were inoculated in a 1:1 ratio in a bioreactor at an OD_600_ of 0.025. A 300 µL sample was removed to measure the initial OD_600_ and the same sample was centrifuged at 15,000 RPM for 3 min. After centrifugation, 250 µL of supernatant was removed and pellets were resuspended in 250 µL of 4% paraformaldehyde in PBS (4% PFA). Samples were kept on ice for 30 min, after which they were centrifuged for 3 min at 15,000 RPM, 250 µL of supernatant were removed, pellets were resuspended in 550 µL PBS, and then stored at 4°C until flow cytometry. After the removal of initial samples (t = 0), 250 µM DPTA NONOate was immediately added to the bioreactor and the ·NO concentration of the reactor was continuously monitored. For subsequent time points, 300 µL was extracted for OD_600_ measurements and the same procedure was used to prepare samples for flow cytometry. Samples were analyzed on an LSRII flow cytometer (BD Biosciences) and the proportion of mCherry positive and GFP-positive cells was quantified. The OD_600_ of the Hmp-proficient subpopulation and the Hmp-deficient subpopulation were calculated by multiplying the total OD_600_ by the respective fraction of fluorescent cells. OD_600_ values were normalized by dividing all values by the initial OD_600_ for each subpopulation. Initial co-culture experiments were performed at an OD_600_ of 0.05 (Fig. S8); however, differences in ·NO detoxification between Hmp-proficient monocultures and cocultures were not large enough to detect differences in growth resumption due to the different time resolutions of those measurements. To address this hurdle, we lowered the initial OD_600_ to 0.025, to better observe differences in ·NO detoxification and growth.

### Heat killing to generate a non-responding population for mixed population experiments

WT harboring pSA21 and Δ*greA*Δ*greB*Δ*araBAD*::P_N25_-*mcherry-gentR* harboring pSA21 were grown to OD_600_ = 0.2 in 20 mL MOPS media with 10 mM glucose supplemented with 50 µg/mL kanamycin, as described above. When OD_600_ reached 0.2, 8 mL of cultures was added to 15 mL centrifuge tubes, and 10 µL was extracted for 10-fold serial dilutions in PBS followed by plating on LB-agar to quantify CFUs/mL prior to heat-killing. Cultures were then incubated in a water bath at ~60°C for 30 min. Afterward, OD_600_ was measured to confirm that appreciable cell lysis had not occurred, and 120 µL was removed to quantify CFUs/mL and confirm cell death. The remaining volume of heat-killed cultures was transferred to microcentrifuge tubes and spun down at 15,000 RPM for 3 min. All supernatants were removed, the cells were concentrated in a total volume of 1 mL PBS, and stored at 4°C prior to use in mixed population experiments.

### Counting non-responding and responding subpopulations

Cells were prepared for inoculation into bioreactors following the same procedure as the ·NO consumption assays, and all cells here contained a low copy plasmid containing an IPTG-inducible P_T5_-*gfp* expression cassette and kanamycin resistance marker for selection (pSA21, Table S1). For both Δ*greA*Δ*greB* and Δ*greA*Δ*greB*Δ*hmp*, cells were inoculated into bioreactors to achieve an OD_600_ of 0.05. For WT live + heat-killed mixed population experiments, live and heat-killed cells were inoculated into bioreactors at a ~2:1 ratio to achieve an OD_600_ of 0.03, which resulted in ·NO clearance times ([·NO] <0.5 μM) similar to that of Δ*greA*Δ*greB* cultures (Fig. S8C). Immediately after bioreactor inoculation, 300 µL was removed to measure OD_600_, and another 300 µL was removed and added to microcentrifuge tubes to be fixed with 4% PFA. DPTA NONOate was added to a concentration of 300 µM and ~30 seconds later 1 mM IPTG was added. Three hundred microliter of samples were taken every 20 min starting at 1 h after the addition of DPTA NONOate until at least 4 h. Samples were fixed for flow cytometry following the same method as the fluorescent reporter assays.

For flow cytometry, fixed samples were diluted with PBS to an OD_600_ ~0.025 in a total volume of 550 µL in flow cytometry tubes. Fifty microliters of AccuCount fluorescent particles (~10^6^ particles/mL, 7.0–7.9 μm nominal size, Spherotech, Inc.) was then added to each sample for a total volume of 600 µL, and they were analyzed on a LSRII flow cytometer. For GFP quantification, the excitation wavelength and emission bandpass filter were set to 488 nm and 525/50. For AccuCount fluorescent particles, which fluoresce in four colors, the excitation wavelength and emission bandpass filter were set to 640 nm and 670/30 to measure APC fluorescence. A sample containing just fluorescent particles (550 µL PBS + 50 µL AccuCount particles), which was called the beads-only sample, was analyzed on the flow cytometer each time. The beads-only sample was used to gate fluorescent particles from forward scatter and APC fluorescence values. Samples were run until ~400 particle events were recorded, which resulted in >40,000 cell events. The total number of cell events in each sample *i* (*N_i_*) was determined from the number of events that fell in the cell gate, which was based on forward scatter and side scatter values. Notably, the beads-only sample had background events in the cell gate that needed to be accounted for. Specifically, the total number of events that fell in the cell gate in the beads-only samples from the same day as experimental samples (*N_beads-only_*) was subtracted from *N_i_* to remove background events contributed by PBS and the fluorescent particle suspension to yield N_i_*.


(1) $$N_{i}^{*}=N_{i}-N_{beads-only}$$

The total cell count per mL of the 600 µL sample (*n_i_*) was calculated as shown below. For samples that were diluted prior to the addition of the fluorescent particles, the cell count was multiplied by the dilution factor to give the total cell count of the original 600 µL fixed sample.


(2) $$n_{i}\left(\frac{\mathrm{cells}}{\mathrm{mL}}\right)=\left[\frac{N_{i}^{*}}{\mathrm{beads}\;\mathrm{in}\;{\mathrm{sample}}_{i}}\times \frac{\mathrm{total}\;\mathrm{number}\;\mathrm{of}\;\mathrm{beads}\;\mathrm{added}}{\mathrm{total}\;\mathrm{volume}\;\mathrm{of}\;\mathrm{sample}\;\left(\mathrm{mL}\right)}\right]\times \left(\mathrm{dilution}\;\mathrm{factor}\right)$$

Next, the fractions of non-responding and responding cells in each sample were calculated according to the equations below. The non-responding cell gates were drawn to include 99 ± 0.02% of the cell events on the GFP fluorescence histograms of samples taken before IPTG induction (t = 0 min on GFP fluorescence histograms). Those non-responding gates were applied to all of the samples from the same experiment to quantify the number of non-responding cell events in each sample (*N(nR)_i_*). To account for background signal from the PBS or particles within the non-responding gate, the number of events within the non-responding gate in the beads-only sample (*N(nR)_beads-only_*) was subtracted from *N(nR)_i_*, and then this quantity was divided by N_i_* to calculate the fraction of non-responding cells in sample *i* (*x(nR)_i_*). A similar calculation was performed for the fraction of responding cells (*x(R)_i_*) in each sample, where responding cell events are all cell events that are not within the non-responding cell gate.


(3) $$x(nR{)}_{i}=\frac{N(nR{)}_{i}-N(nR{)}_{\text{beads-only }}}{N_{i^{*}}}$$

(4) $$x(R{)}_{i}=\frac{\left[N_{i}-N(nR{)}_{i}\right]-\left[N_{\text{beads-only }}-N(nR{)}_{\text{beads-only }}\right]}{N_{i^{*}}}$$

The non-responding and responding cell counts for each sample (*n(nR)_i_* and *n(R)_i_*, respectively) were calculated as shown below. These counts were absolute cells/mL and are portrayed in Fig. S8J through O.


(5) $$n{\left(nR\right)}_{i}\left(\frac{cells}{mL}\right)=\;x{\left(nR\right)}_{i}\;\times \;n_{i}$$

(6) $$n{\left(R\right)}_{i}\left(\frac{\text{cells}}{\text{mL}}\right)=\;x{\left(R\right)}_{i}\;\times \;n_{i}$$

For some plots (Fig. 12; Fig. S8D through I), non-responding and responding cell counts were normalized relative to their values at the time point closest to but not beyond clearance, which is designated as t = 0 min after clearance on the normalized cell count plots (*i* = 0).


(7) $$\text{ Normalized cell count of nR}\text{ population of sample i}=\frac{n(nR{)}_{i}}{n(nR{)}_{0}}$$

(8) $$Normalized\;cell\;count\;of\;R\;population\;of\;sample\;i=\frac{n{\left(R\right)}_{i}}{n{\left(R\right)}_{0}}$$

### Sorting and co-stress experiments with Δ*greA*Δ*greB* and WT

Cells were prepared for inoculating bioreactors following the same method as the ·NO consumption assays. For experiments with live non-responding populations, Δ*greA*Δ*greB*Δ*araBAD*::P_N25_-*mcherry-gentR* harboring pSA21 was inoculated into a bioreactor at OD_600_ = 0.02. This strain constitutively expresses mCherry. Immediately after inoculation, 300 µL were removed to measure OD_600_ and a second 300 µL of sample was fixed with 4% PFA as a control for flow cytometry to ensure that GFP expression had not been initiated prior to IPTG induction. The bioreactor was immediately treated with 300 µM DPTA NONOate and then 30 seconds later 1 mM IPTG was added. WT harboring pUA66 was inoculated into a separate bioreactor ~30 min later at OD_600_ = 0.01. The lower density was used to enable ·NO stress to persist in the culture while the non-responsive cells of Δ*greA*Δ*greB*Δ*araBAD*::P_N25_-*mcherry-gentR* with pSA21 were sorted by FACS. Immediately after inoculation, 300 µL were removed to measure OD_600_, the culture was treated with 300 µM DPTA NONOate, and [·NO] was monitored continuously. Forty-five minutes after treating Δ*greA*Δ*greB*Δ*araBAD*::P_N25_-*mcherry-gentR* with pSA21 with DPTA NONOate, 2 mL of the culture was added to a flow cytometry tube, and the sample was kept at 37°C. Two million low-GFP cells were sorted on a FACS Aria Fusion cell-sorter at 37°C in PBS, where low-GFP cells were collected from the lower half of the low-GFP peak. Immediately after the conclusion of sorting, ~300 µM DPTA NONOate was added to the sorted sample to maintain ·NO stress, and the sample was kept at 37°C. Then, 1 mM IPTG was added to WT bioreactors for consistency with the Δ*greA*Δ*greB* bioreactors and 300 µL samples were removed and fixed with 4% PFA. Those samples were used to define the mCherry negative gates for flow cytometry. Then sorted low-GFP cells (~2 mL) were immediately added to the bioreactor. After 15 min (0.25 h), to allow time for mixing, 300 µL samples were removed for fixation with 4% PFA. Five hours later (5.25 h), the second samples were removed for fixation, and [·NO] monitoring concluded because the concentration had reached the baseline. Terminal samples were removed 6 hours later (11.25 h) and fixed. For experiments with heat-killed cells, heat-killed Δ*greA*Δ*greB*Δ*araBAD*::P_N25_-*mcherry-gentR* harboring pSA21 were used as the non-responding population. WT harboring pUA66 were inoculated into bioreactors following the same method as for the live non-responder experiments. The heat-killed cells were diluted to OD_600_ ~0.025 in 2.6 mL PBS. Three hundred microliter of samples were removed to measure OD_600_ and for fixation with 4% PFA to assess mCherry fluorescence with flow cytometry. The heat-killed cells were maintained at 37°C. The times at which DPTA NONOate was added to the heat-killed cells in PBS and the heat-killed cells were inoculated into the WT bioreactor were based on the times at which these steps were performed for the sorted low-GFP non-responding cells. The same sampling and IPTG treatment were performed prior to inoculation of heat-killed cells as was done for the sorted non-responding cells, and then heat-killed non-responding cells were inoculated into the WT bioreactor. Samples were taken and fixed at 0.25 h, 5.25 h, and 11.25 h following the same protocols as the sorted low-GFP cells. All samples were fixed following the same method as the fluorescent reporter assays.

Fixed samples were prepared for flow cytometry following the same method as for counting non-responding and responding populations and analyzed on an LSRII flow cytometer. Fifty microliters of AccuCount fluorescent particles were added to samples taken from the WT bioreactor right before the addition of the non-responders and to all samples from the bioreactor after the addition of non-responders. GFP and mCherry fluorescence were monitored using the excitation wavelengths and emission bandpass filters described for the absorbance and fluorescence measurements, and APC fluorescence of AccuCount fluorescent particles was measured using the same parameters as for counting non-responding and responding populations. Fluorescent particle events were gated from forward scatter vs APC fluorescence using a beads-only sample, and ~5,000 fluorescent particle events were recorded for each sample. Cell events were gated from forward scatter vs side scatter. The total number of mCherry negative cell events was determined by gating ≥99.99% of the cell events in the mCherry vs side scatter plot for the sample taken immediately before the addition of the non-responding cells to the WT bioreactor. This gate was then applied to the samples taken after inoculation of the mCherry positive (non-responding) cells. All events with higher mCherry fluorescence were considered mCherry positive. Similar to equation (1), the number of total cell events and number of mCherry negative cell events in the beads-only sample from each flow cytometry experiment were subtracted from the number of total cell events and number of mCherry negative cell events in each experimental sample, respectively, to correct for background signal from the particles or PBS. The corrected number of mCherry-positive events was calculated as the corrected total number of cell events less the corrected number of mCherry-negative cell events. This was converted to a mCherry-positive cell count (cells/mL) following equation (2), and mCherry-positive cell counts at 5.25 and 11.25 h were normalized by the mCherry-positive cell counts at 0.25 h post-inoculation.

### Statistical analyses

Statistical significance was assessed with *t*-tests with unequal variances for comparisons of two samples, whereas one-way ANOVA followed by post hoc Tukey HSD tests were used for comparison of three samples or more. A *P*-value threshold <0.05 was considered statistically significant.

## SUPPLEMENTAL MATERIAL

Figure S1 mbio.02229-24-s0001.tif Experiments for complementation, culturability, unstressed translation, and unstressed growth and requirements for hmp and translation during ·NO stress.

Figure S2 mbio.02229-24-s0002.tif Complementation of *greA* or *greB* restores unimodal fluorescence from the *hmp* promoter.

Figure S3 mbio.02229-24-s0003.tif ·NO detoxification for Δ*hmp* and Δ*greA*Δ*greB*Δ*hmp* cells expressing Hmp-GFP translational fusions under the T5 promoter.

Figure S4 mbio.02229-24-s0004.tif Sorting culturability and subpopulation growth characteristics.

Figure S5 mbio.02229-24-s0005.tif tdBroccoli fluorescence measurements in the absence of ·NO.

Figure S6 mbio.02229-24-s0006.tif GFP expression from the T7 promoter requires T7 RNA polymerase.

Figure S7 mbio.02229-24-s0007.tif GFP distributions at lower H_2_O_2_ concentrations and culturability measurements at 75 µM H_2_O_2_.

Figure S8 mbio.02229-24-s0008.pdf Additional data from experiments to assess cheating.

Table S1 mbio.02229-24-s0009.pdf List of bacterial strains and plasmids.

Table S2 mbio.02229-24-s0010.pdf Oligonucleotides used in this study.

ASM does not own the copyrights to Supplemental Material that may be linked to, or accessed through, an article. The authors have granted ASM a non-exclusive, world-wide license to publish the Supplemental Material files. Please contact the corresponding author directly for reuse.
