# Supplementary figures and images for: Loss of Gre factors leads to phenotypic heterogeneity and cheating in Escherichia coli populations under nitric oxide stress
Source: mBio. 2024 Sep 9;15(10):e02229-24. doi: 10.1128/mbio.02229-24 (PMC11498084; doi:10.1128/mbio.02229-24)

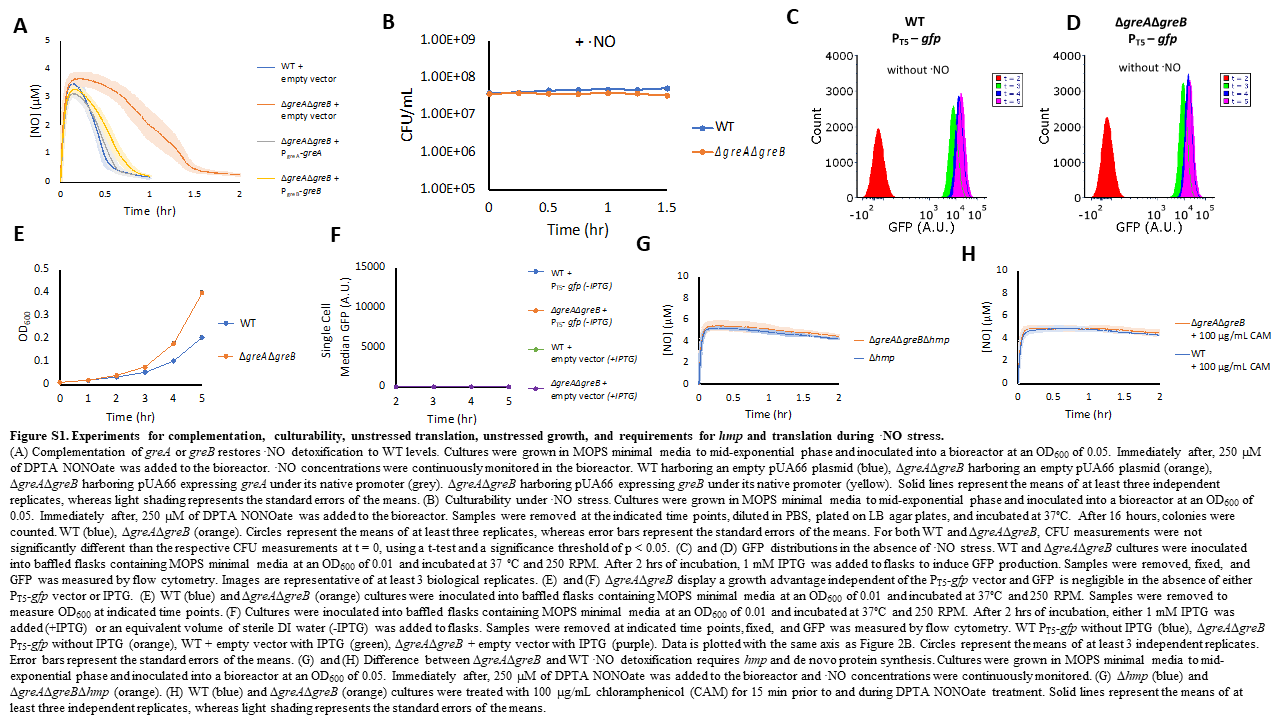

Supplement: Figure S1 — Experiments for complementation, culturability, unstressed translation, and unstressed growth and requirements for hmp and translation during ·NO stress. [file mbio.02229-24-s0001.tif]

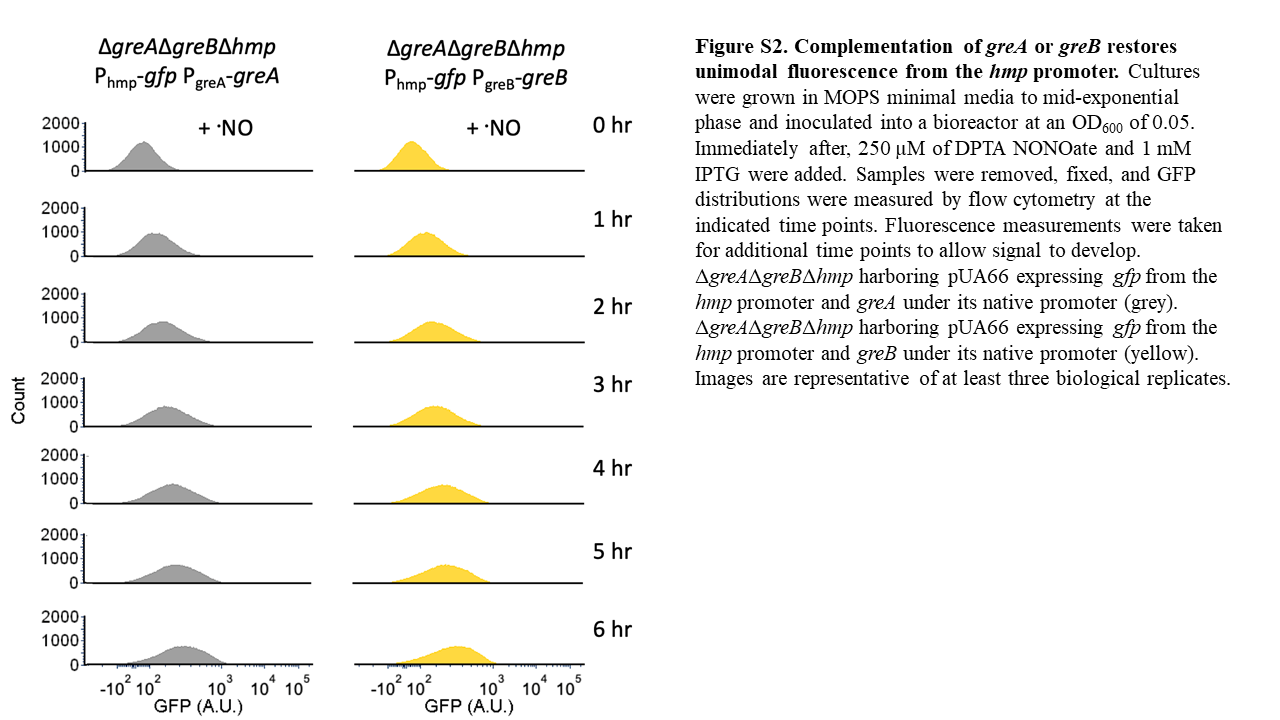

Supplement: Figure S2 — Complementation of greA or greB restores unimodal fluorescence from the hmp promoter. [file mbio.02229-24-s0002.tif]

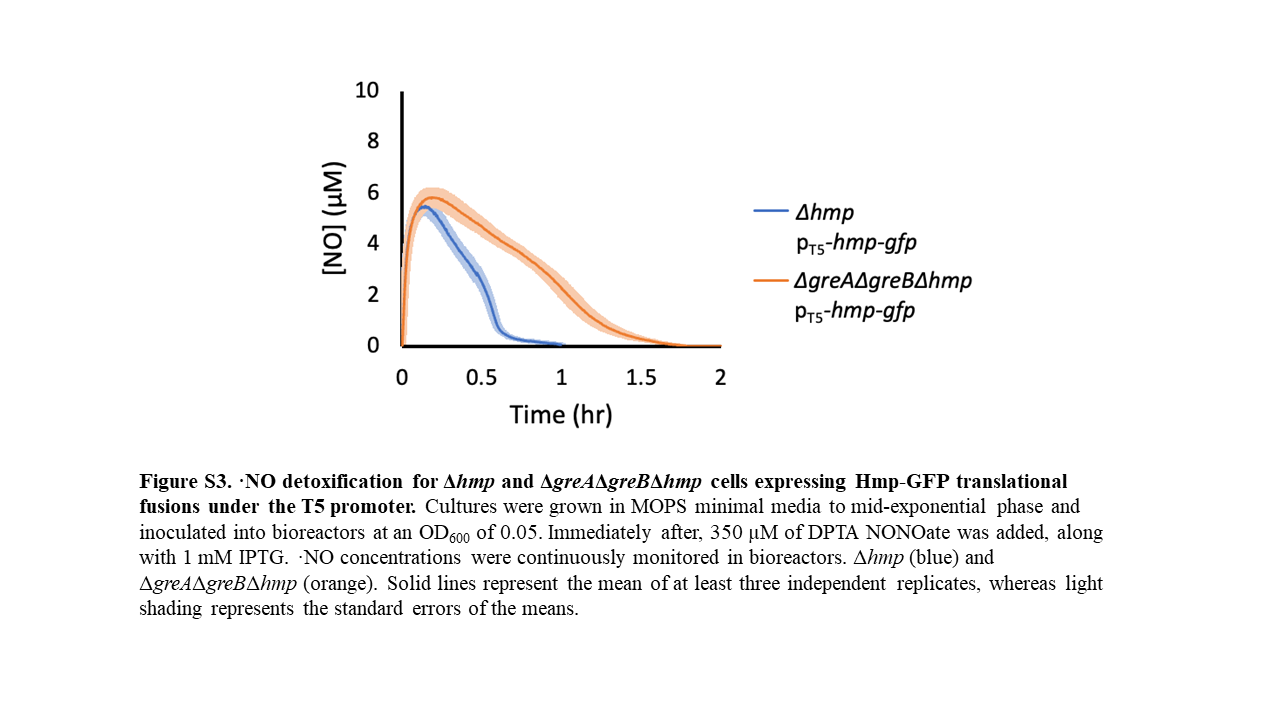

Supplement: Figure S3 — ·NO detoxification for Δhmp and ΔgreAΔgreBΔhmp cells expressing Hmp-GFP translational fusions under the T5 promoter. [file mbio.02229-24-s0003.tif]

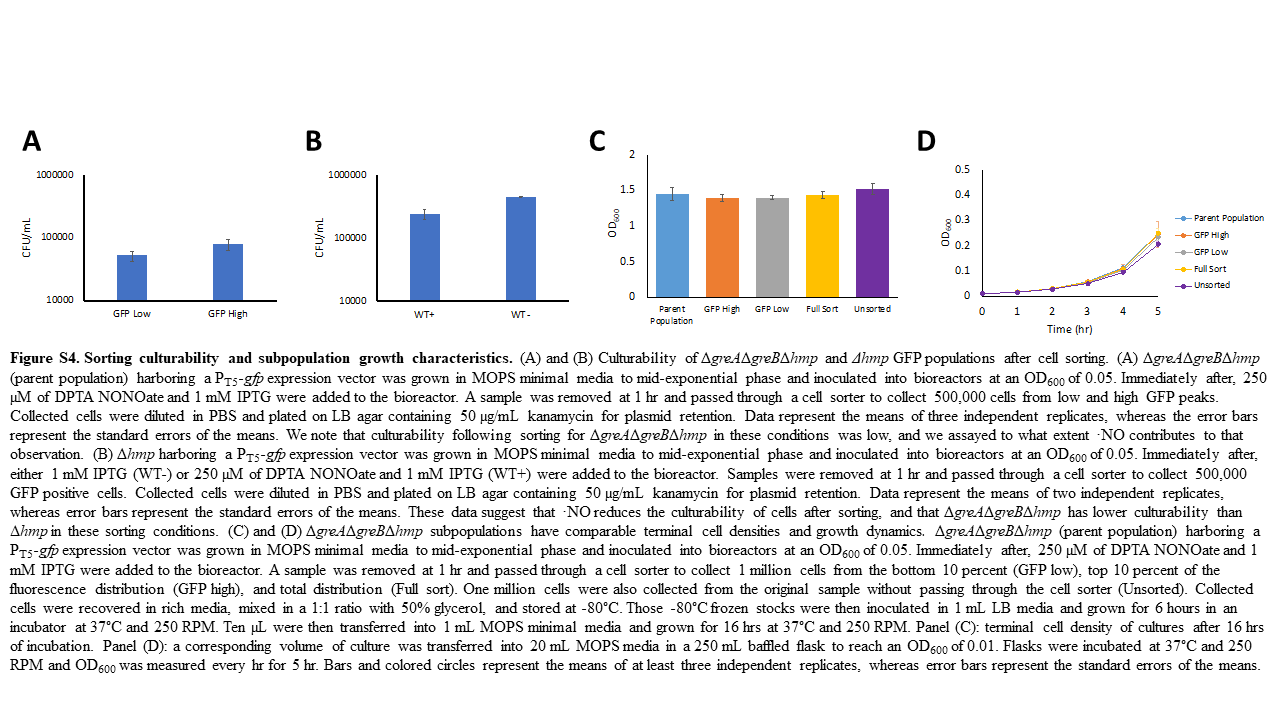

Supplement: Figure S4 — Sorting culturability and subpopulation growth characteristics. [file mbio.02229-24-s0004.tif]

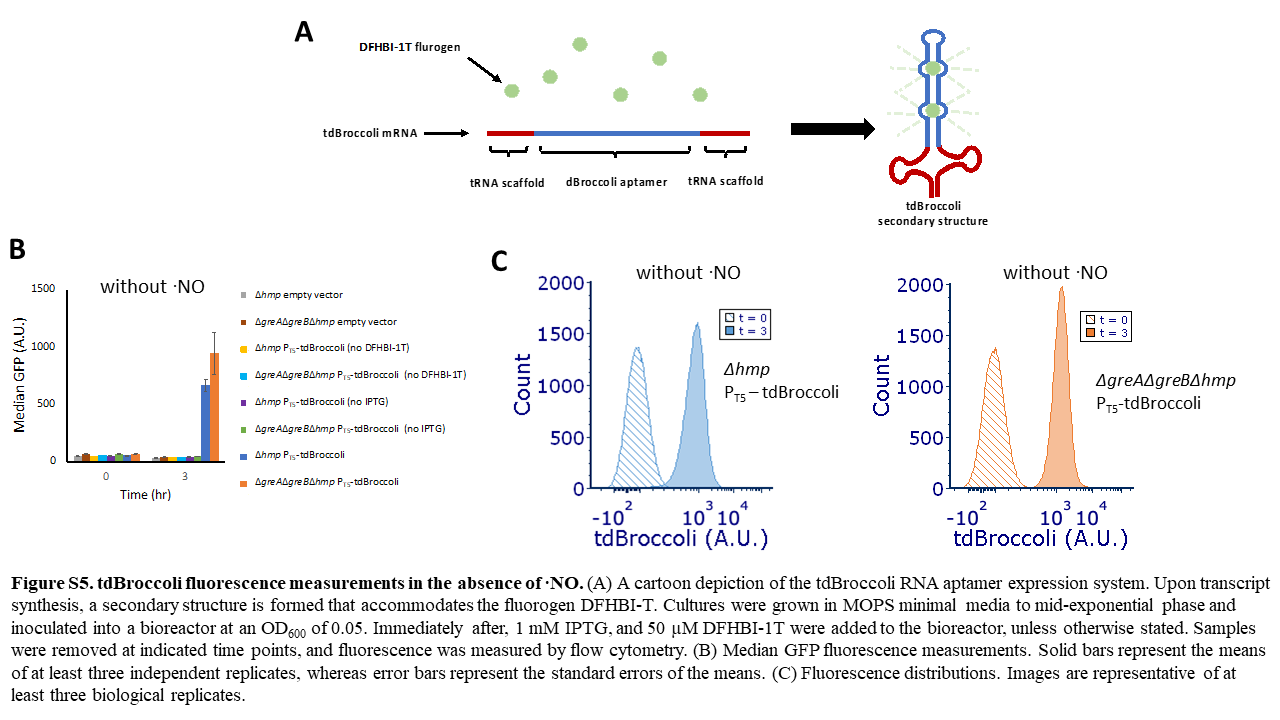

Supplement: Figure S5 — tdBroccoli fluorescence measurements in the absence of ·NO. [file mbio.02229-24-s0005.tif]

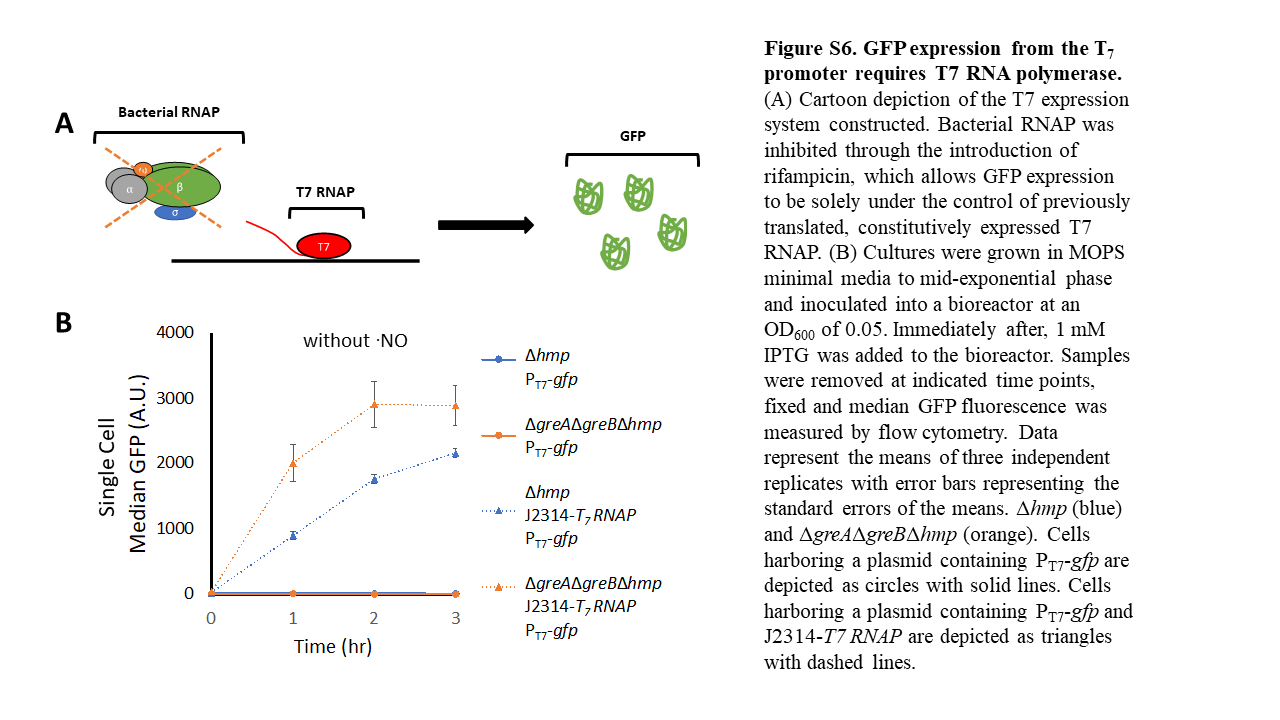

Supplement: Figure S6 — GFP expression from the T7 promoter requires T7 RNA polymerase. [file mbio.02229-24-s0006.tif]

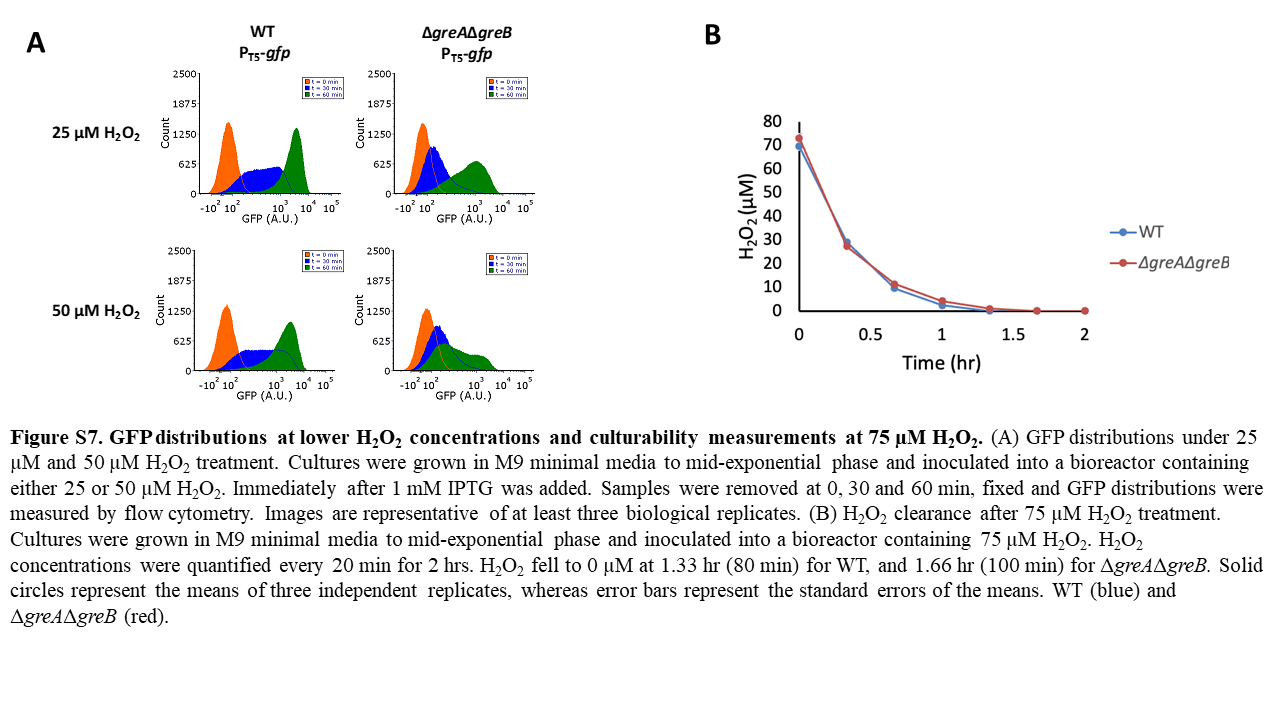

Supplement: Figure S7 — GFP distributions at lower H2O2 concentrations and culturability measurements at 75 µM H2O2. [file mbio.02229-24-s0007.tif]
