# Supplementary material for: Loss of Gre factors leads to phenotypic heterogeneity and cheating in Escherichia coli populations under nitric oxide stress
Source: mBio. 2024 Sep 9;15(10):e02229-24. doi: 10.1128/mbio.02229-24 (PMC11498084; doi:10.1128/mbio.02229-24)
Supplement: Figure S8 — Additional data from experiments to assess cheating. [file mbio.02229-24-s0008.pdf]

Fig S8

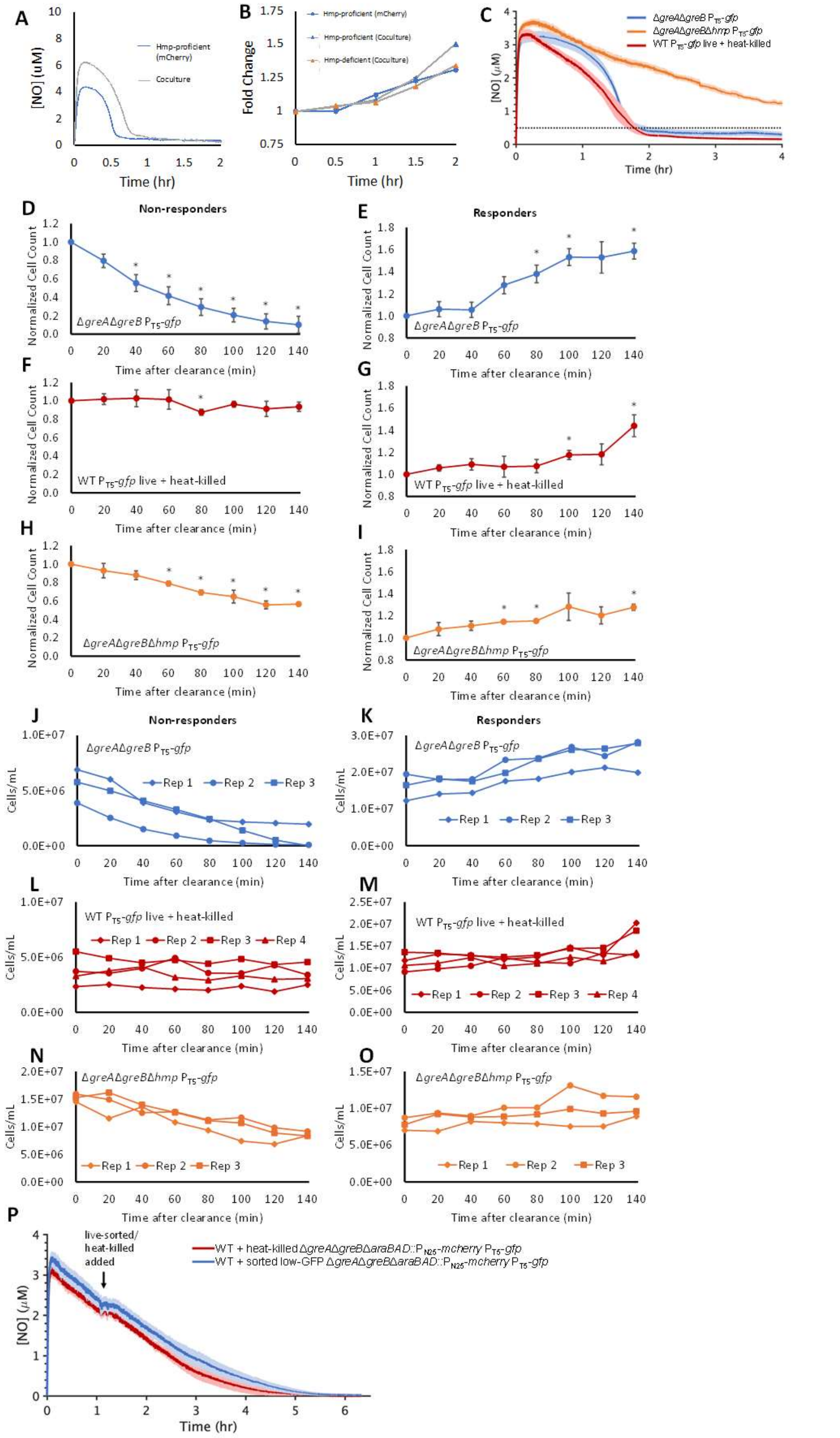

Figure S8. Additional data from experiments to assess cheating. (A-B) Cultures were grown in MOPS minimal media to mid-exponential phase and inoculated into a bioreactor at an OD_{600} of 0.05 either as a monoculture of Hmp-proficient cells (blue) or as a 1:1 coculture of Hmp-proficient cells and Hmp-deficient cells (grey). Immediately after, 250 μM of DPTA NONOate was added to the bioreactor. (A) ·NO concentrations were continuously monitored in the bioreactor. Solid lines represent a single replicate. (B) Samples were removed to measure OD_{600} at indicated time points. For cocultures, samples were fixed and the OD_{600} was scaled by the proportion of GFP and mCherry fluorescent cells estimated by flow cytometry. Colored circles and triangles represent a single replicate. (C) [·NO] was monitored continuously in bioreactors of *ΔgreAΔgreB* (blue), *ΔgreAΔgreBΔhmp* (orange), and WT live + heat-killed cocultures (red). Solid lines represent the means of at least three independent replicates, whereas light shading represents the standard errors of the means. (D) Normalized *ΔgreAΔgreB* P_{T5}-*gfp* non-responding cell counts decrease significantly after ·NO clearance. (E) Normalized *ΔgreAΔgreB* P_{T5}-*gfp* responding cell counts increase significantly after ·NO clearance. (F) Normalized WT live + heat-killed coculture nonresponding cell counts do not change appreciably after 140 min. (G) Normalized WT live + heat-killed coculture responding cell counts increase significantly within 140 min after ·NO clearance. (H) Normalized *ΔgreAΔgreBΔhmp* P_{T5}-*gfp* nonresponding cell counts decrease modestly over time. (I) Normalized *ΔgreAΔgreBΔhmp* P_{T5}-*gfp* responding cell counts increase significantly over time. Data points show the mean of at least three independent replicates, whereas errors bars represent the standard error of the means. Asterisks indicate statistical significance between data at specific time points and t=0 min, at a p-value ≤ 0.05, which was assessed by t-tests. (J) Absolute *ΔgreAΔgreB* P_{T5}-*gfp* non-responding cell counts. (K) Absolute *ΔgreAΔgreB* P_{T5}-*gfp* responding cell counts. (L) Absolute WT live + heat-killed coculture nonresponding cell counts. (M) Absolute WT live + heat-killed coculture responding cell counts. (N) Absolute *ΔgreAΔgreBΔhmp* P_{T5}-*gfp* nonresponding cell counts. (O) Absolute *ΔgreAΔgreBΔhmp* P_{T5}-*gfp* responding cell counts. (P) [·NO] was monitored continuously in bioreactors of cocultures of WT with live-sorted (blue) or WT with heat-killed (red) *ΔgreAΔgreB* added at the indicated time. Solid lines represent the means of at least three independent replicates, whereas light shading represents the standard errors of the means.
