# Supplementary material for: Loss of Gre factors leads to phenotypic heterogeneity and cheating in Escherichia coli populations under nitric oxide stress
Source: mBio. 2024 Sep 9;15(10):e02229-24. doi: 10.1128/mbio.02229-24 (PMC11498084; doi:10.1128/mbio.02229-24)
Supplement: Table S1 — List of bacterial strains and plasmids. [file mbio.02229-24-s0009.pdf]

Table S1. List of bacterial strains and plasmids.

Strain	Genotype	Notes
**MG1655**	F⁻ λ⁻ *ilvG⁻ rfb-50 rph-1*	ATCC 700926 [1]
**MO001**	MG1655 Δ*lacI::lacIq* Δ*lacZYA*::P_{T5}- *mcherry-kanR*	[2]
**MO002**	MG1655 Δ*lacI::lacIq* Δ*lacZYA*::P_{T5}- *mcherry*	[3]
**WM020**	MG1655 Δ*lacI::lacIq* Δ*lacZYA*::P_{T5}- *gfp-kanR*	[4]
**DS001**	MG1655 Δ*hmp::kanR*	P1 transduction of mutation in Keio collection into MG1655
**DS002**	MG1655 Δ*greA::kanR*	P1 transduction of mutation in Keio collection into MG1655
**DS003**	MG1655 Δ*greB::kanR*	P1 transduction of mutation in Keio collection into MG1655
**DS004**	MG1655 Δ*hmp*	*kanR* cured from DS001
**DS005**	MG1655 Δ*greA*	*kanR* cured from DS002
**DS006**	MG1655 Δ*greB*	*kanR* cured from DS003
**DS007**	MG1655 Δ*greB*Δ*greA::kanR*	P1 transduction of mutation in Keio collection into DS003
**DS008**	MG1655 Δ*greB*Δ*greA*	*kanR* cured from DS007
**DS009**	MG1655 Δ*greB*Δ*greA*Δ*hmp::kanR*	P1 transduction of mutation in Keio collection into DS008
**DS010**	MG1655 Δ*greB*Δ*greA*Δ*hmp*	*kanR* cured from DS009
**DS011**	MG1655 Δ*lacI::kanR*	*lacI* deleted with lambda red system
**DS012**	MG1655 Δ*lacI*	*kanR* cured from DS011
**DS013**	MG1655 Δ*lacI* Δ*hmp::kanR*	P1 transduction of mutation in Keio collection into DS012
**DS014**	MG1655 Δ*lacI*Δ*hmp*	*kanR* cured from DS013
**DS013**	MG1655 Δ*lacI*Δ*araBAD*::P_{T5}-*mcherry-kanR*	P_{T5}-*mcherry-kanR* knocked into DS012 with lambda red system. Used as Hmp-proficient strain in coculture.
**DS014**	MG1655 Δ*lacI*Δ*hmp*Δ*araBAD*::P_{T5}-*gfp-kanR*	P_{T5}-*gfp-kanR* knocked into DS014 with lambda red system. Used as Hmp-deficient strain in coculture.
**DS015**	MG1655 Δ*greB*Δ*greA*Δ*araBAD*::P_{N25}-*mcherry-gentR*	P_{N25}-*mcherry-gentR* knocked into DS008 with lambda red system.
**Plasmid**	Genotype	Source
**pUA66**	Vector, SC101ori, *kanR*, *gfpmut2* reporter	[5]
**pJR05**	pUA66 P_{T5}-*hmp-gfp*$_{sf}$, *lacIq*	[1]
**pSA21**	pUA66 P_{T5}-*gfp*$_{sf}$, *lacIq*	[6]
**pXW02**	pUA66 P_{hmp}-*gfp*$_{sf}$	[7]
**pXW09**	pQE80 P_{T5}-*gfp*$_{sf}$, *lacIq*	[7]
**pTOX66**	pUA66 P_{N25}-*tetR-gentR*, P_{LtetO1}-*mazF*	[3]
**pDS01**	pUA66 P_{greA}-*greA*	BamHI SbfI restriction digest and ligation
**pDS02**	pUA66 P_{greB}-*greB*	BamHI SbfI restriction digest and ligation
**pDS03**	pUA66 P_{T7}-*gfp*, *lacIq*	Hifi DNA assembly
**pDS04**	pUA66 J23114 – *T7RNAP*, P_{T7}-*gfp*, *lacIq*	Hifi DNA assembly
**pDS05**	pQE80 P_{T5}- tdBroccoli, *lacIq*	Hifi DNA assembly
**pDS06**	pQE80 P_{T5}- *mcherry*- tdBroccoli , *lacIq*	Hifi DNA assembly
**pDS07**	pUA66 P_{hmp}-*gfp*$_{sf,}$ P_{greA}-*greA*	Hifi DNA assembly
**pDS08**	pUA66 P_{hmp}-*gfp*$_{sf,}$ P_{greB}-*greB*	Hifi DNA assembly
**pDS09**	pTOX66 P_{N25}-*mcherry-gentR*	Gibson assembly

References

1. Robinson JL, Brynildsen MP. An ensemble-guided approach identifies ClpP as a major regulator of transcript levels in nitric oxide-stressed Escherichia coli. Metab Eng. 2015;31:22–34.
2. Orman MA, Brynildsen MP. Dormancy is not necessary or sufficient for bacterial persistence. Antimicrob Agents Chemother. 2013;57(7):3230–9.
3. Mok WW, Park JO, Rabinowitz JD, Brynildsen MP. RNA futile cycling in model Persisters derived from MAZF accumulation. mBio. 2015;6(6).
4. Mok WW, Brynildsen MP. Timing of DNA damage responses impacts persistence to fluoroquinolones. Proc Natl Acad Sci. 2018;115(27).
5. Zaslaver A, Bren A, Ronen M, Itzkovitz S, Kikoin I, Shavit S, et al. A comprehensive library of fluorescent transcriptional reporters for escherichia coli. Nat Methods. 2006;3(8):623–8.
6. Amato SM, Brynildsen MP. Persister heterogeneity arising from a single metabolic stress. Curr Biol. 2015;25(16):2090–8.
7. Wan X, Brynildsen MP. Robustness of nitric oxide detoxification to nitrogen starvation in Escherichia coli requires RelA. Free Radic Biol Med. 2021;176:286–97.
