# Supplementary material for: Loss of Gre factors leads to phenotypic heterogeneity and cheating in Escherichia coli populations under nitric oxide stress
Source: mBio. 2024 Sep 9;15(10):e02229-24. doi: 10.1128/mbio.02229-24 (PMC11498084; doi:10.1128/mbio.02229-24)
Supplement: Table S2 — Oligonucleotides used in this study. [file mbio.02229-24-s0010.pdf]

Table S2. Oligonucleotides used in this study

Mutant Construction	Forward primer	Reverse primer	Description
ΔlacI::kanR	TCTGGTGGCCGGAAGGCGAAGCGGCATGCATTTACGTTGAGTGTAGGCTGGAGCTGCTTC	TGCCTAATGAGTGAGCTAACTCACATTAATTGCGTTGCGCCATATGAATATCCTCCTTAG	Used to amplify kanR with FRT sites from pKD13, and the linear DNA was used with lambda Red system to replace lacI on the chromosome.
ΔaraBAD::P_{T5}-gfp-kanR	GCCATAGCATTTTTATCCATAAGATTAGCGGATCCTACCTCTCGAGAAATCATAAAAAAT	AGCCTGGTTTCGTTTGATTGGCTGTGGTTTTATACAGTCATCAGAAGAACTCGTCAAGAA	Used to amplify P_{T5}-gfp-kanR from WM020, and the linear DNA was used with lambda Red system to replace araBAD on the chromosome.
ΔaraBAD::P_{T5}-mcherry-kanR	GCCATAGCATTTTTATCCATAAGATTAGCGGATCCTACCTCTCGAGAAATCATAAAAAAT	AGCCTGGTTTCGTTTGATTGGCTGTGGTTTTATACAGTCATCAGAAGAACTCGTCAAGAA	Used to amplify P_{T5}-mcherry-kanR from MO001, and the linear DNA was used with lambda Red system to replace araBAD on the chromosome.
ΔaraBAD::P_{N25}-mcherry-gentR	GCCATAGCATTTTTATCCATAAGATTAGCGGATCCTACCTTCATAAAAAATTTATTTGCTTTCAGGA	AGCCTGGTTTCGTTTGATTGGCTGTGGTTTTATACAGTCATTAGGTGGCGGTACTTGG	Used to amplify P_{N25}-mcherry-gentR from pDS09, and the linear DNA was used with lambda Red system to replace araBAD on the chromosome.

Table S2A. Primer sequences for mutant construction

Mutant Verification	External Forward	External Reverse	Internal Forward	Internal Reverse	**Description**
Δhmp	TTTATAGGCGGTCCTGTTGG	AGGCGACCTTTTCAAGGAAT	TAACCGCCCATTTCTACGAC	TACGATAGCCTTTGCCATCC	Absence of locus was confirmed by cPCR using the following combinations of primers:<br>1) External Forward, External Reverse<br>2) Internal Forward, Internal Reverse<br>3) External Forward, kanR Internal Reverse
ΔgreA	GTACCCGGGTCGGTATTTTT	CCACGCTCTGTTCGTTGATA	CCACGCTCTGTTCGTTGATA	ACAGCTTGGCTTCGATGTCT	Absence of locus was confirmed by cPCR using the following combinations of primers:<br>1) External Forward, External Reverse<br>2) Internal Forward, Internal Reverse;<br>3) External Forward, kanR Internal Reverse
ΔgreB	CTCACGTTCGTCACCAGCTA	CATCGGCAGGAGGTTAAGAC	AAAAAGCGTCTGCGTGAAAT	TCGGGGAATCGATAGAGATG	Absence of locus was confirmed by cPCR using the following combinations of primers:<br>1) External Forward, External Reverse<br>2) Internal Forward, Internal Reverse<br>3) External Forward, kanR Internal Reverse
ΔlacI	GGGATCAGGAGGAGAAGATCG		ATCGAATGGCGCAAAACCT	TTCCAGTCGGGAAACCTGT	Absence of locus was confirmed by cPCR using the following combinations of primers:<br>1) Internal Forward, Internal Reverse<br>2) External Forward, kanR Internal Reverse
ΔaraBAD::P_{T5}-gfp/mcherry-kanR		TCAGCCAGACAAACGTTGAG	ATGATTGAACAAGATGGATTGC		Absence of locus was confirmed by cPCR using the following combinations of primers:<br>1) Internal Forward, Internal Reverse
ΔaraBAD::P_{N25}-mcherry-gentR	CGTTAATGATTTGCCCAAAC		CAGAAAGGGCAATTTTGTGATGC	CAACCTGACCGCAAATACCT	Absence of locus was confirmed by cPCR using the following combinations of primers<br>1) Internal Forward, Internal Reverse<br>2) External Forward, araBAD::P_{T5}-gfp External Reverse<br>3) External Forward, gentR Internal Reverse
kanR				GAAGCGGTCAGCCCATTC	
gentR				CGACCGAAAAGATCAAGAGC	

Table S2B. Primer sequences for mutant verification

Plasmid Construction	Genotype	Forward primer	Reverse primer	Description
pDS01	pUA66 P_{greA}-greA	GAGGGGATCCACGATGACCCTTCGGGAACT	CATGCCTGCAGGGGTAATTCTTACAGGTATTC	Amplify P_{greA}-greA from MG1655 genomic DNA. Digest pUA66 and amplicon with BamHI and SbfI. Perform ligation with the Quick Ligation Kit (NEB).
pDS02	pUA66 P_{greB}-greB	GAGGGGATCCCAGCTTAAGTATACAATTTA	CATGCCTGCAGGTCTTCCTTACGGTTTCACGT	Amplify P_{greB}-greB from MG1655 genomic DNA. Digest pUA66 and amplicon with BamHI and SbfI. Perform ligation with the Quick Ligation Kit (NEB).
pDS03	puA66 P_{T7}-gfp, lacIq	GGGTTTTTTGCCAAGCTAGCTTGGCGAG	AGTCGTATTATTTCTCGAGCGATGACGTC	Amplify pSA21 with Phusion to be used in Hifi DNA assembly reaction. Double stranded DNA insert (P_{T7}-gfp) was purchased from Integrated DNA Technologies.
pDS04	pUA66 J23114 – T_7RNAP, P_{T7}-gfp, lacIq	CAAGAAAAGCCCGTCACGGGCTTC	TATTATGGGTAGTTTCCTTGCATGAATCCA	Amplify pDS03 with Phusion to be used in Hifi DNA assembly reaction. Double stranded DNA insert (J23114-T_7RNAP) was purchased from Integrated DNA Technologies.
pDS05	pQE80 P_{T5}-tdBroccoli, lacIq	CCTATTTCCCTAAAGGGTTTATTGAGAATATGTTTTTC	TTGAATCTATTATAATTGTTATCCGCTCACAAAGCAAATAAATTT	Amplify pXW09 with Phusion to be used in Hifi DNA assembly reaction. Double stranded DNA insert (tdBroccoli) was purchased from Integrated DNA Technologies.
pDS06	pQE80 P_{T5}-mcherry-tdBroccoli , lacIq	CTCTACGACAACCTCTTCACAGCCAATCTCGCCCGGATAGCTCAGTC	GCAAATAAATTTTTTATGATTTCTCGAG	Amplify pDS05 with Phusion to be used in Hifi DNA assembly reaction with mcherry.
		CTCGAGAAATCATAAAAAATTTATTTGC	GAGATTGGCTGTGAAGAGGTTGTCGTAGAGCTACTTGTACAGCTCGTCCA	Amplify mcherry from MO002 genomic DNA.
pDS07	pUA66 P_{hmp}-gfpsf, P_{greA}-greA	CAAGAAAAGCCCGTCACGGGCTTC	TATTATGGGTAGTTTCCTTGCATGAATCCA	Amplify pXW02 with Phusion to be used in Hifi DNA assembly reaction. Double stranded DNA insert (P_{greA}-greA) was purchased from Integrated DNA Technologies.
pDS08	pUA66 P_{hmp}-gfpsf, P_{greB}-greB	CAAGAAAAGCCCGTCACGGGCTTC	TATTATGGGTAGTTTCCTTGCATGAATCCA	Amplify pXW02 with Phusion to be used in Hifi DNA assembly reaction. Double stranded DNA insert (P_{greB}-greB) was purchased from Integrated DNA Technologies.
pDS09	pUA66 P_{N25}-mcherry-gentR, P_{LtetO1}-mazF	AAGGTCGATGTTTGATGTTATG	AATGAATCTATTATACAGAAAAATTTTCC	Amplify pTOX66 with Phusion to be used in Gibson assembly reaction with *mcherry*.
		TTCTGTATAATAGATTCATTAAAGAGGAGAAATTAACTATGGTG	TAACATCAAACATCGACCTTCTACTTGTACAGCTCGTCCATGC	Amplify *mcherry* from DS013 genomic DNA.

Table S2C. Primer sequences for plasmid construction

Insert	Sequence
pDS03 insert	TAATACGACTCACTATAGATTCAATTGTGAGCGGATAACAATTTCACACAGAATTCATTAAAGAGGAGACCTCTAGTATGAGCAAAGGAGAAGAACTTTTCACTGGAGTTGTCCCAATTCTTGTTGAATTAGATGGTGATGTTAATGGGCACAAATTTTCTGTCCGTGGAGAGGGTGAAGGTGATGCTACAAACGGAAAACTCACCCTTAAATTTATTTGCACTACTGGAAAACTACCTGTTCCGTGGCCAACACTTGTCACTACTCTGACCTATGGTGTTCAATGCTTTTCCCGTTATCCGGATCACATGAAACGGCATGACTTTTTCAAGAGTGCCATGCCCGAAGGTTATGTACAGGAACGCACTATATCTTTCAAAGATGACGGGACCTACAAGACGCGTGCTGAAGTCAAGTTTGAAGGTGATACCCTTGTTAATCGTATCGAGTTAAAGGGTATTGATTTTAAAGAAGATGGAAACATTCTTGGACACAAACTCGAGTACAACTTTAACTCACACAATGTATACATCACGGCAGACAAACAAAAGAATGGAATCAAAGCTAACTTCAAAATTCGCCACAACGTTGAAGATGGTTCCGTTCAACTAGCAGACCATTATCAACAAAATACTCCAATTGGCGATGGCCCTGTCCTTTTACCAGACAACCATTACCTGTCGACACAATCTGTCCTTTCGAAAGATCCCAACGAAAAGCGTGACCACATGGTCCTTCTTGAGTTTGTAACTGCTGCTGGGATTACACATGGCATGGATGAGCTCTACAAATAAGGATCCGCATGCGAGCTCGGTACCCCGGGTCGACCTGCAGCCAAGCTTAATTAGCTGAGCTTGTAGCATAACCCCTTGGGGCCTCTAAACGGGTCTTGAGGGGTTTTTTG
pDS04 insert	CAAGGAAACTACCCATAATAtttatggctagctcagtcctaggtacaatgctagcTTCACACAGAATTCATTAAAGAGGAGAAATTAACTatgAACACGATTAACATCGCTAAGAACGACTTCTCTGACATCGAACTGGCTGCTATCCCGTTCAACACTCTGGCTGACCATTACGGTGAGCGTTTAGCTCGCGAACAGTTGGCCCTTGAGCATGAGTCTTACGAGATGGGTGAAGCACGCTTCCGCAAGATGTTTGAGCGTCAACTTAAAGCTGGTGAGGTTGCGGATAACGCTGCCGCCAAGCCTCTCATCACTACCCTACTCCCTAAGATGATTGCACGCATCAACGACTGGTTTGAGGAAGTGAAAGCTAAGCGCGGCAAGCGCCCGACAGCCTTCCAGTTCCTGCAAGAAATCAAGCCGGAAGCCGTAGCGTACATCACCATTAAGACCACTCTGGCTTGCCTAACCAGTGCTGACAATACAACCGTTCAGGCTGTAGCAAGCGCAATCGGTCGGGCCATTGAGGACGAGGCTCGCTTCGGTCGTATCCGTGACCTTGAAGCTAAGCACTTCAAGAAAAACGTTGAGGAACAACTCAACAAGCGCGTAGGGCACGTCTACAAGAAAGCATTTATGCAAGTTGTCGAGGCTGACATGCTCTCTAAGGGTCTACTCGGTGGCGAGGCGTGGTCTTCGTGGCATAAGGAAGACTCTATTCATGTAGGAGTACGCTGCATCGAGATGCTCATTGAGTCAACCGGAATGGTTAGCTTACACCGCCAAAATGCTGGCGTAGTAGGTCAAGACTCTGAGACTATCGAACTCGCACCTGAATACGCTGAGGCTATCGCAACCCGTGCAGGTGCGCTGGCTGGCATCTCTCCGATGTTCCAACCTTGCGTAGTTCCTCCTAAGCCGTGGACTGGCATTACTGGTGGTGGCTATTGGGCTAACGGTCGTCGTCCTCTGGCGCTGGTGCGTACTCACAGTAAGAAAGCACTGATGCGCTACGAAGACGTTTACATGCCTGAGGTGTACAAAGCGATTAACATTGCGCAAAACACCGCATGGAAAATCAACAAGAAAGTCCTAGCGGTCGCCAACGTAATCACCAAGTGGAAGCATTGTCCGGTCGAGGACATCCCTGCGATTGAGCGTGAAGAACTCCCGATGAAACCGGAAGACATCGACATGAATCCTGAGGCTCTCACCGCGTGGAAACGTGCTGCCGCTGCTGTGTACCGCAAGGACAAGGCTCGCAAGTCTCGCCGTATCAGCCTTGAGTTCATGCTTGAGCAAGCCAATAAGTTTGCTAACCATAAGGCCATCTGGTTCCCTTACAACATGGACTGGCGCGGTCGTGTTTACGCTGTGTCAATGTTCAACCCGCAAGGTAACGATATGACCAAAGGACTGCTTACGCTGGCGAAAGGTAAACCAATCGGTAAGGAAGGTTACTACTGGCTGAAAATCCACGGTGCAAACTGTGCGGGTGTCGATAAGGTTCCGTTCCCTGAGCGCATCAAGTTCATTGAGGAAAACCACGAGAACATCATGGCTTGCGCTAAGTCTCCACTGGAGAACACTTGGTGGGCTGAGCAAGATTCTCCGTTCTGCTTCCTTGCGTTCTGCTTTGAGTACGCTGGGGTACAGCACCACGGCCTGAGCTATAACTGCTCCCTTCCGCTGGCGTTTGACGGGTCTTGCTCTGGCATCCAGCACTTCTCCGCGATGCTCCGAGATGAGGTAGGTGGTCGCGCGGTTAACTTGCTTCCTAGTGAAACCGTTCAGGACATCTACGGGATTGTTGCTAAGAAAGTCAACGAGATTCTACAAGCAGACGCAATCAATGGGACCGATAACGAAGTAGTTACCGTGACCGATGAGAACACTGGTGAAATCTCTGAGAAAGTCAAGCTGGGCACTAAGGCACTGGCTGGTCAATGGCTGGCTTACGGTGTTACTCGCAGTGTGACTAAGCGTTCAGTCATGACGCTGGCTTACGGGTCCAAAGAGTTCGGCTTCCGTCAACAAGTGCTGGAAGATACCATTCAGCCAGCTATTGATTCCGGCAAGGGTCTGATGTTCACTCAGCCGAATCAGGCTGCTGGATACATGGCTAAGCTGATTTGGGAATCTGTGAGCGTGACGGTGGTAGCTGCGGTTGAAGCAATGAACTGGCTTAAGTCTGCTGCTAAGCTGCTGGCTGCTGAGGTCAAAGATAAGAAGACTGGAGAGATTCTTCGCAAGCGTTGCGCTGTGCATTGGGTAACTCCTGATGGTTTCCCTGTGTGGCAGGAATACAAGAAGCCTATTCAGACGCGCTTGAACCTGATGTTCCTCGGTCAGTTCCGCTTACAGCCTACCATTAACACCAACAAAGATAGCGAGATTGATGCACACAAACAGGAGTCTGGTATCGCTCCTAACTTTGTACACAGCCAAGACGGTAGCCACCTTCGTAAGACTGTAGTGTGGGCACACGAGAAGTACGGAATCGAATCTTTTGCACTGATTCACGACTCCTTCGGTACCATTCCGGCTGACGCTGCGAACCTGTTCAAAGCAGTGCGCGAAACTATGGTTGACACATATGAGTCTTGTGATGTACTGGCTGATTTCTACGACCAGTTCGCTGACCAGTTGCACGAGTCTCAATTGGACAAAATGCCAGCACTTCCGGCTAAAGGTAACTTGAACCTCCGTGACATCTTAGAGTCGGACTTCGCGTTCGCGtaaGGATCCGCATGCGAGCTCGGTACCCCGGGTCGACCTGCAGCCAAGCTTAATTAGCTGAGCTTGGACTCCTGTTGATAGATCCAGTAATGACCTCAGAACTCCATCTGGATTTGTTCAGAACGCTCGGTTGCCGCCGGGCGTTTTTTATTGGTGAGAATCAAGAAAAGCCCGTCACGGG
pDS05 insert	AACAATTATAATAGATTCAATTGTGAGCGGATAACAATTTCGCCCGGATAGCTCAGTCGGTAGAGCAGCGGAGACGGTCGGGTCCATCTGAGACGGTCGGGTCCAGATATTCGTATCTGTCGAGTAGAGTGTGGGCTCAGATGTCGAGTAGAGTGTGGGCTCCGCGGGTCCAGGGTTCAAGTCCCTGTTCGGGCGCCAGACTCCTGTTGATAGATCCAGTAATGACCTCAGAACTCCATCTGGATTTGTTCAGAACGCTCGGTTGCCGCCGGGCGTTTTTTATTGGTGAGAATCCAAGCTAGCTTGGCGAGATTTTCAGGAGCTAAGGAAGCTAAAATGGAGAAAAAAATCACTGGATATACCACCGTTGATATATCCCAATGGCATCGTAAAGAACATTTTGAGGCATTTCAGTCAGTTGCTCAATGTACCTATAACCAGACCGTTCAGCTGGATATTACGGCCTTTTTAAAGACCGTAAAGAAAAATAAGCACAAGTTTTATCCGGCCTTTATTCACATTCTTGCCCGCCTGATGAATGCTCATCCGGAATTTCGTATGGCAATGAAAGACGGTGAGCTGGTGATATGGGATAGTGTTCACCCTTGTTACACCGTTTTCCATGAGCAAACTGAAACGTTTTCATCGCTCTGGAGTGAATACCACGACGATTTCCGGCAGTTTCTACACATATATTCGCAAGATGTGGCGTGTTACGGTGAAAACCTGGCCTATTTCCCTAAAGGGTTT
pDS07 insert	CAAGGAAACTACCCATAATAGATCCACGATGACCCTTCGGGAACTTCAGGGTAAAATGACTATCAAAATGTGAATTGTAGCTGACCTGGGACTTGTACCCGGGTCGGTATTTTTTTGCTTCTGGTCCCGGTAAGGAGTTATGCCGGGCAGGCCGAACAGCCGGGGTGGGTGAAGACTTGCCCTATCAGGAATATTCAAGAGGTATAACAAatgCAAGCTATTCCGATGACCTTACGCGGCGCTGAAAAATTACGCGAAGAGCTGGATTTTCTGAAATCTGTGCGCCGTCCTGAAATCATTGCTGCTATCGCGGAAGCGCGTGAGCATGGCGACCTGAAAGAAAACGCCGAATACCACGCAGCTCGTGAACAGCAGGGTTTCTGCGAAGGCCGTATTAAAGACATCGAAGCCAAGCTGTCGAACGCGCAGGTGATTGATGTCACCAAAATGCCCAACAATGGGCGCGTTATTTTTGGTGCTACCGTAACGGTGCTGAATCTGGATTCTGACGAAGAACAGACTTATCGCATCGTTGGCGATGACGAAGCTGACTTTAAACAAAACCTGATTTCTGTAAACTCGCCTATTGCTCGTGGCCTGATCGGCAAAGAAGAAGATGATGTTGTGGTCATCAAAACGCCGGGCGGCGAAGTAGAATTTGAAGTAATTAAGGTGGAATACCTGtaaGAATTACCCCTGCAGGCATGCAAGCTCTAGAGGCATCAAATAAAACGAAAGGCTCAGTCGAAAGACTGGGCCTTTCGTTTTATCTGTTGTTTGTCGGTGAACGCTCTCCTGAGTAGGACAAATCAAGAAAAGCCCGTCACGGG
pDS08 insert	CAAGGAAACTACCCATAATAGATCCCAGCTTAAGTATACAATTTATTCGGCGAAACATTATTGATTCTGTTGATATGATCACGTTATACCCAATGTGCGCATTATCAAACAGACAAAGGGAATCAACGAGatgAAAACGCCCCTGGTTACCCGGGAAGGGTATGAAAAACTCAAACAAGAGCTTAATTATCTCTGGCGTGAAGAACGCCCGGAGGTCACAAAAAAGGTGACCTGGGCCGCAAGTCTGGGCGACCGCAGCGAAAATGCTGACTATCAGTATAATAAAAAGCGTCTGCGTGAAATCGACCGTCGCGTGCGCTATCTCACTAAATGCCTGGAAAATCTCAAAATCGTCGATTACTCCCCTCAGCAGGAAGGCAAAGTCTTTTTTGGCGCGTGGGTGGAGATTGAAAACGACGATGGCGTGACTCACCGTTTCCGTATTGTCGGCTACGATGAAATTTTTGGCCGTAAAGATTACATCTCTATCGATTCCCCGATGGCCCGCGCATTGCTGAAAAAAGAAGTCGGCGATCTGGCGGTGGTGAATACCCCTGCCGGGGAAGCGAGCTGGTATGTTAATGCTATCGAGTACGTGAAACCGtaaGGAAGACCTGCAGGCATGCAAGCTCTAGAGGCATCAAATAAAACGAAAGGCTCAGTCGAAAGACTGGGCCTTTCGTTTTATCTGTTGTTTGTCGGTGAACGCTCTCCTGAGTAGGACAAATCAAGAAAAGCCCGTCACGGG

Table S2D. Double stranded insert sequences
